# scDMV: a zero–one inflated beta mixture model for DNA methylation variability with scBS-seq data

**DOI:** 10.1093/bioinformatics/btad772

**Published:** 2023-12-23

**Authors:** Yan Zhou, Ying Zhang, Minjiao Peng, Yaru Zhang, Chenghao Li, Lianjie Shu, Yaohua Hu, Jianzhong Su, Jinfeng Xu

**Affiliations:** School of Mathematical Sciences, Institute of Statistical Sciences, Shenzhen Key Laboratory of Advanced Machine Learning and Applications, Shenzhen University, Shenzhen, China; School of Mathematical Sciences, Institute of Statistical Sciences, Shenzhen Key Laboratory of Advanced Machine Learning and Applications, Shenzhen University, Shenzhen, China; School of Mathematics and Statistics and KLAS, Northeast Normal University, Changchun, China; School of Biomedical Engineering, School of Ophthalmology & Optometry and Eye Hospital, Wenzhou Medical University, Wenzhou, China; School of Biomedical Engineering, School of Ophthalmology & Optometry and Eye Hospital, Wenzhou Medical University, Wenzhou, China; Faculty of Business Administration, University of Macau, Macau, China; School of Mathematical Sciences, Institute of Statistical Sciences, Shenzhen Key Laboratory of Advanced Machine Learning and Applications, Shenzhen University, Shenzhen, China; School of Biomedical Engineering, School of Ophthalmology & Optometry and Eye Hospital, Wenzhou Medical University, Wenzhou, China; Department of Biostatistics, City University of Hong Kong, Tat Chee Avenue, Hong Kong, China

## Abstract

**Motivation:**

The utilization of single-cell bisulfite sequencing (scBS-seq) methods allows for precise analysis of DNA methylation patterns at the individual cell level, enabling the identification of rare populations, revealing cell-specific epigenetic changes, and improving differential methylation analysis. Nonetheless, the presence of sparse data and an overabundance of zeros and ones, attributed to limited sequencing depth and coverage, frequently results in reduced precision accuracy during the process of differential methylation detection using scBS-seq. Consequently, there is a pressing demand for an innovative differential methylation analysis approach that effectively tackles these data characteristics and enhances recognition accuracy.

**Results:**

We propose a novel beta mixture approach called scDMV for analyzing methylation differences in single-cell bisulfite sequencing data, which effectively handles excess zeros and ones and accommodates low-input sequencing. Our extensive simulation studies demonstrate that the scDMV approach outperforms several alternative methods in terms of sensitivity, precision, and controlling the false positive rate. Moreover, in real data applications, we observe that scDMV exhibits higher precision and sensitivity in identifying differentially methylated regions, even with low-input samples. In addition, scDMV reveals important information for GO enrichment analysis with single-cell whole-genome sequencing data that are often overlooked by other methods.

**Availability and implementation:**

The scDMV method, along with a comprehensive tutorial, can be accessed as an R package on the following GitHub repository: https://github.com/PLX-m/scDMV.

## 1 Introduction

Epigenetics explores heritable changes in gene expression independent of DNA sequence alterations (Dupont *et al.* 2009). Key epigenetic modifications such as DNA methylation, histone modification, promoter–enhancer interaction, and noncoding RNA regulation play crucial roles and can contribute to diseases (Allis and Jenuwein 2016, Chen *et al.* 2017). Among these modifications, DNA methylation has gained significant attention due to its reversible nature and potential as a therapeutic target (Baylin and Jones 2011, Ahuja *et al.* 2014, Maresca and Wismayer 2016, Mohamad *et al.* 2019). In mammals, DNA methylation primarily occurs at CpG sites, where the fifth carbon atom of cytosine is methylated by DNA methyltransferase, resulting in 5-methylcytosine (Bird 1987, Kulis and Esteller 2010). CpG sites can be dispersed throughout the DNA sequence or concentrated in CpG islands within regulatory regions (Hodges *et al.* 2009). Understanding DNA methylation is essential in elucidating its impact on cell development, disease progression, and gene regulation (Takai and Jones 2002, Das and Singal 2004, Altun *et al.* 2010, Bock *et al.* 2012, Smith and Meissner 2013, Khavari *et al.* 2014, Sheaffer *et al.* 2014, Stelzer *et al.* 2016, Koch *et al.* 2018).

Analyzing differential DNA methylation between samples is crucial for understanding disease etiology, aiding in disease prevention and diagnosis. Two common approaches for differential methylation analysis are differentially methylated site (DMS) analysis and differentially methylated region (DMR) analysis. DMS analysis focuses on individual methylation sites within a single sample and is less directly linked to gene expression. In contrast, DMR analysis considers contiguous regions comprising one or more DMSs and allows for comparisons across multiple sample groups, providing more insights into gene expression.

In recent years, there has been a proliferation of sequencing-based methods for identifying differential methylation. These methods incorporate a wide range of approaches such as logistic regression, beta-binomial distribution, hidden Markov models, Shannon entropy, and binary segmentation smoothing. Existing algorithms include “eDMR” (2013) (Li *et al.* 2013), “RADMeth” (2014) (Dolzhenko and Smith 2014), “BSmooth” (2014) (Hansen *et al.* 2012), and “CGmapTools” (2018) (Guo *et al.* 2018).

The use of traditional strategies for studying DNA methylation diversity is limited when relying on averaged data from multiple cells. Single-cell whole-genome bisulfite sequencing (scWGBS and scRRBS) has emerged as a promising approach to assess DNA methylation diversity in individual cells and rare cell types. However, the sparsity and unique characteristics of single-cell DNA methylation sequencing data, including low coverage and excess zeros and ones (e.g. the sum of the methylation rates at 0 and 1 exceeds 0.9 in the real example of Section 2.4), render traditional statistical methods inadequate. Therefore, novel methods are needed to perform differential methylation analysis using scBS-seq data.

In this paper, we propose a strategy called scDMV (zero–one inflated beta mixture model) for analyzing single-cell bisulfite data. We assume that the scBS-seq data, conditioned on the cell and region-specific effect representing the methylation rate, follow a binomial distribution. In addition, we model the effect distribution using a zero–one inflated beta distribution to account for the excess of zeros and ones, as well as the over-dispersion observed in scBS-seq data. We employ the EM algorithm to estimate the model parameters and utilize the Wald test for conducting differential methylation analysis. We compare the performance of scDMV with two existing methods, methylpy and CGmapTools, through numerical studies including simulation experiments and real data applications. The results demonstrate the superior performance of scDMV, particularly in capturing important information for GO enrichment analysis using single-cell whole-genome sequencing data.

## 2 Materials and methods

The observed scBS-seq data consist of a collection denoted by

$$\left(n_{\mathrm{gij}},x_{\mathrm{gij}}\right),\;g=1,\;2;\;i=1,\ldots ,N_{g};\;j=1,\ldots ,M,$$

where $\left.n_{\mathrm{gij}}\right.$ represents the total reads obtained from the ith cell of type g in region j, $x_{\mathrm{gij}}$ represents the methylation reads acquired from the ith cell of type g in region j, g corresponds to the cell type; $N_{g}$ indicates the number of cells belonging to type g, i represents the samples or cells, j pertains to different CG regions, and M represents the total number of CG segments considered.

Let $p_{gj}$ represents the methylation rate of cells belonging to type g in region j. We define $P_{g}$ as the vector of methylation rates for type g cells, given by $P_{g}={\left(p_{g1},\ldots ,p_{gM}\right)}^{T}$. The primary objective of differential methylation analysis is to examine whether the null hypothesis of equal average methylation levels between two methylation rate vectors holds. The alternative hypothesis, denoted as $H_{1}$, suggests the presence of specific regions, denoted as $m\in \left\{1,\ldots ,M\right\}$, where the average methylation rates differ between the two cell types. To address this hypothesis testing problem, we first construct a test statistic. Subsequently, we develop a procedure to identify the set of DMRs.

### 2.1 Formulating the model and test statistic

Given the total reads $n_{\mathrm{gij}}$, it is reasonable to assume that the count of methylation reads $x_{\mathrm{gij}}$ follows a binomial distribution, which can be expressed as:

Pxgijngij,pgj=ngijxgijpgjxgij(1-pgj)ngij-xgij,

where $p_{gj}$ represents the methylation rate of cells of type g in region j. It is important to note that the methylation rate of cells shows significant heterogeneity, often characterized by an excess of zeros and ones. To capture this variability, we model $p_{gj}$ as a random effect and define its mean as $Ep_{gj}=\mu_{gj}$. For each region j, we test the hypothesis

$$H_{0j}\;:\;\mu_{1j}=\mu_{2j}\;\mathrm{versus}\;H_{aj}\;:\;\mu_{1j}\neq \mu_{2j}.$$

Let $x_{gj}=\sum_{i=1}^{n_{g}}x_{\mathrm{gij}}$, $x_{j}=\sum_{g=1}^{2}x_{gj}$, $n_{gj}=\sum_{i=1}^{n_{g}}n_{\mathrm{gij}}$ and $n_{j}=\sum_{g=1}^{2}n_{gj}$. Given the observation that the DNA methylation rate $p_{gj}$ in single cells tends to concentrate around values of 0 and 1, we assume that $p_{gj}$ follows a mixed beta distribution with 0–1 inflation. This distribution can be characterized as follows:

f(pgj)= πgj0, pgj=0  πgj1, pgj=11-πgj0-πgj1Betaαgj,βgj, pgjϵ0,1. 

Within this model, the assessment of differential methylation expression between two types of samples reduces to the examination of:

$${\hat{H}}_{0j}:{\left(\pi_{1j0},\pi_{1j1},\alpha_{1j},\beta_{1j}\right)}^{T}={\left(\pi_{2j0},\pi_{2j1},\alpha_{2j},\beta_{2j}\right)}^{T}.$$

It is important to emphasize that the four parameters associated with the two types of samples can vary across CG regions j. Now, let us proceed to derive the algorithm for estimating these parameters. For a given value of g and j, we calculate the likelihood function and utilize the EM algorithm to estimate the parameter $\theta_{g}=\left(\pi_{gj0},{\;\pi }_{gj1},\;\alpha_{gj},\;\beta_{gj}\right)$. The information matrix $I\left(\pi_{gj0},\;\pi_{gj1},\;\alpha_{gj},{\;\beta }_{gj}\right)$ takes the form

$$\;E\left(\begin{array}{cc} \begin{array}{cc} \frac{\partial^{2}\log L}{\partial \pi_{gj0}^{2}} & \frac{\partial^{2}\log L}{\partial \pi_{gj0}\partial \pi_{gj1}}\\ \frac{\partial^{2}\log L}{\partial \pi_{gj1}\partial \pi_{gj0}} & \frac{\partial^{2}\log L}{\partial \pi_{gj1}^{2}} \end{array} & \begin{array}{cc} 0\; & 0\\ 0\; & 0 \end{array}\\ \begin{array}{cc} 0 & \;0\\ 0 & \;0 \end{array} & \begin{array}{cc} \frac{\partial^{2}\log L}{\partial \alpha_{gj}^{2}} & \frac{\partial^{2}\log L}{\partial \alpha_{gj}\partial \beta_{gj}}\\ \frac{\partial^{2}\log L}{\partial \beta_{gj}\partial \alpha_{gj}} & \frac{\partial^{2}\log L}{\partial \beta_{gj}^{2}} \end{array} \end{array}\right)$$

and the expected value of the methylation rate of the type g cells in region j

$$\left.g\left(\theta_{g}\right)\right.=Ep_{gj}=\pi_{gj1}+\left(1-\pi_{gj0}-\pi_{gj1}\right)\frac{\alpha_{gj}}{\alpha_{gj}+\beta_{gj}}.$$

**Figure 1.**
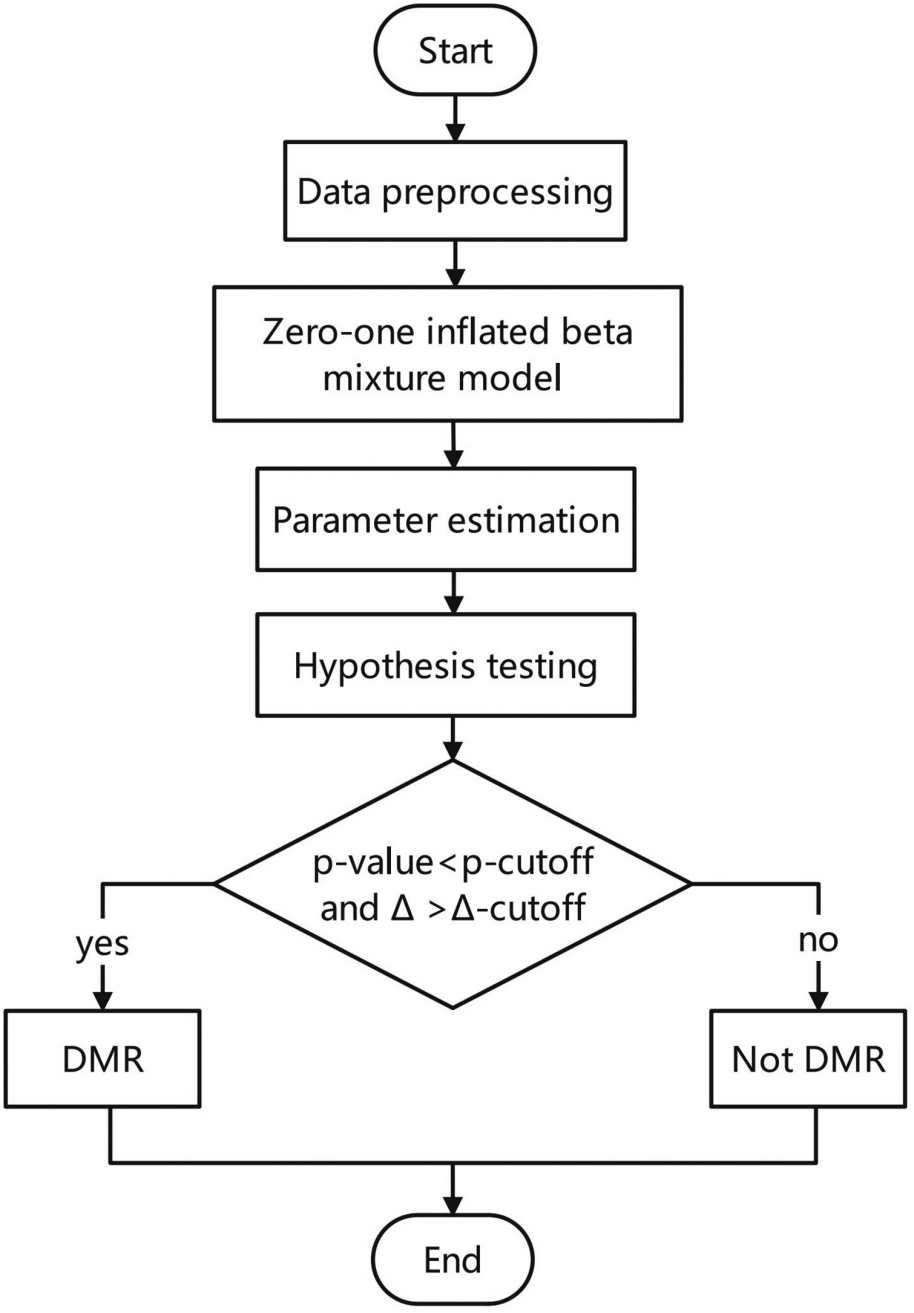
Flowchart of scDMV. The flowchart represents the scDMV method, comprising the following steps: (A) data input and preprocessing, (B) apply the propose model, (C) parameter estimation, (D) hypothesis testing, and (E) calculate the corresponding *P*-value and Δ and compare with the cutoffs.

Under the null hypothesis, the resulting Wald test statistic

(1) $$T=\frac{g\left({\hat{\theta }}_{1}\right)-g\left({\hat{\theta }}_{2}\right)}{\sqrt{{\left(\frac{\partial g\left(\theta_{1}\right)}{\partial \theta_{1}}\right)}^{T}I\left(\theta_{1}\right)\frac{\partial g\left(\theta_{1}\right)}{\partial \theta_{1}}+{\left(\frac{\partial g\left(\theta_{2}\right)}{\partial \theta_{2}}\right)}^{T}I\left(\theta_{2}\right)\frac{\partial g\left(\theta_{2}\right)}{\partial \theta_{2}}}}$$

asymptotically follows a standard normal distribution. In summary, the test statistic is derived based on Equation (1), and the flow chart in Fig. 1 illustrates the scDMV method.

### 2.2 DMR identification

Next, we evaluate all M regions to obtain M *P*-values. Regions with *P*-values below the specified cutoff are considered significantly different and classified as DMRs. Subsequently, commonly used methods leverage the DNA methylation differences among samples to refine the initially obtained DMRs for enhanced accuracy. In our study, the weighted DNA methylation is employed to calculate the methylation degree difference, denoted as Δ, between the two types of samples within each region. A cutoff value is then set for Δ, and the final DMRs must satisfy the condition that Δ exceeds the cutoff. Generally, the DMRs identified by the model are those regions with *P*-values below the *P*-value cutoff and Δ exceeding the Δ cutoff.

### 2.3 Simulation study

The existing methods, CGmapTools and methylpy, are specifically designed to analyze differences in cell clusters formed by a large number of cells using traditional bisulfite sequencing technologies. Consequently, they may not be directly applicable to single-cell data. In contrast, the novel statistical method proposed in this study, called scDMV, takes into account the specific characteristics of low coverage and depth in scBS-seq sequencing data. To assess and compare the performance of CGmapTools, methylpy, and the new scDMV method in identifying DMRs in single-cell DNA methylation data, we conducted several simulation experiments based on simulated scBS-seq data.

We generated a simulated dataset consisting of 73 samples, with two distinct cell types having sample sizes of 48 and 25, respectively. Each sample comprised 10 000 sites, where each site had methylation reads represented by x and total reads denoted by n. The dataset was divided into 1000 regions, with every 10 consecutive sites forming a region. The data simulation process involved three main steps. Firstly, we assigned values to the four parameters $\left(\pi_{10},\pi_{11},\alpha_{1},\beta_{1}\right)$ and $\left(\pi_{20},\pi_{21},\alpha_{2},\beta_{2}\right)$ for the two sample types. Secondly, we obtained simulated total reads based on the actual total reads. Finally, the simulated methylation reads were generated according to the underlying theoretical model.

To illustrate the process of generating simulated data, we consider a specific region as an example. Firstly, we generate the simulated total reads data, denoted as n, by randomly sampling 10 nonzero values from each column in the true total read data. Subsequently, the methylation reads x for that region are generated based on the prior distribution mentioned earlier. By following this procedure, we obtain the simulated data for a particular region.

The aforementioned process is repeated 1000 times to generate simulation data for 1000 regions. The methylation reads x and total reads n data for each region are stored in separate lists, resulting in a collection of 1000 lists. These 1000 lists are then combined into a single comprehensive list, representing the final simulation data. The sum of the methylation rates at 0 and 1 in the simulated data is ∼0.66.

Two types of simulation experiments were conducted: difference experiments and indifference experiments. In the difference experiments, the parameter values $\left(\pi_{10},\pi_{11},\alpha_{1},\beta_{1}\right)$ and $\left(\pi_{20},\pi_{21},\alpha_{2},\beta_{2}\right)$ were deliberately set to be different, resulting in simulated data that exhibited differences between the groups. On the other hand, in the indifference experiments, $\left(\pi_{10},\pi_{11},\alpha_{1},\beta_{1}\right)$ and $\left(\pi_{20},\pi_{21},\alpha_{2},\beta_{2}\right)$ were set to be exactly the same, generating simulated data with no group differences. For each type of experiment, five sets of experimental data were simulated.

To assess the comparative accuracy of the scDMV method in identifying DMRs, we conducted simulation experiments and compared its performance with methylpy and CGmapTools. The regional average methylation level of a sample group was defined as the ratio of the sum of methylation reads to the sum of total reads for all sites in that region across all samples within the group. The difference in methylation level, denoted as Δ, represented the disparity in regional average methylation levels between the two sample groups within the same region. For each method, *P*-values and Δ values were calculated for each region separately. We employed different cutoff values for the *P*-value (0.001, 0.005, 0.01, and 0.05) and Δ (0, 0.1, 0.15, and 0.2). Regions satisfying both the *P*-value not exceeding the specified cutoff and Δ surpassing the defined threshold were considered as identified DMRs.

### 2.4 Real data applications

To explore the methylation patterns during early embryonic development, we employed the scDMV method on a publicly available dataset (Benjamini and Hochberg 1995) and compared its outcomes with two alternative methods.

#### 2.4.1 Design of real data experiments

The dataset (GEO ID: GSE81233) consists of 73 samples from two consecutive developmental stages, comprising 25 4-cell samples and 48 8-cell samples. Both within-group and between-group experiments were conducted. In the between-group experiment, which involved samples with differences, we utilized all 73 samples to identify DMRs between the 25 4-cell embryo samples and the 48 8-cell embryo samples. The experimental results from the three methods were obtained by applying various *P*-value cutoffs and different thresholds for the difference in DNA methylation level (Δ). In the within-group experiment, where there were no sample differences, we selected 40 out of the 48 8-cell embryo samples and equally divided them into two groups to identify DMRs. Since the overall methylation patterns tend to remain stable across samples of the same developmental stage (Benjamini and Hochberg 1995), there should be no individual variations in methylation between the two groups. Consequently, the identified DMRs in this scenario can be considered as false-positive DMRs.

In order to enhance the accuracy of identifying DMRs, we performed data preprocessing and filtering procedures. Initially, we assessed the significance of each site to determine its impact on the region. Sites with a missing value exceeding 50% of the total number of samples were excluded. After site filtering, the data were segmented into regions with a maximum length of 300 bp, ensuring that each region contained a minimum of 3 sites. Following the aforementioned data organization approach, each region was represented by two lists: one for the total reads data and another for the methylation reads data. All regions of each chromosome were stored as a collective list called “testRegion,” thereby forming a list for each chromosome. Subsequently, the data within “testRegion” were utilized for conducting experiments using the scDMV method. During the experiments, we employed a weighted approach to calculate the regional methylation levels. We executed the experiments on each chromosome, generating *P*-values and Δ values for each region. Finally, we applied the cutoffs defined earlier to filter the regions accordingly.

In the final step, we combined the outcomes from both experiments to conduct a comprehensive analysis and comparison of the three methods, thereby assessing the performance of the scDMV method in identifying DMRs.

#### 2.4.2 Annotation of DMRs

The DMRs identified between the 8-cell stage and 4-cell stage were annotated using the ChIPSeeker R package (version 1.24.0) (Yu *et al.* 2015), based on their corresponding regions in the human genome (hg19). The annotation process involved classifying the DMRs based on their locations relative to gene transcription, including promoter regions (within 2 kb from the Transcription Start Site or TSS), introns, exons, and intergenic regions. In addition, the DMRs were annotated based on their association with CpG islands, CpG shores (within 2 kb from an island), CpG shelves (within 2 kb from a shore), and the open sea (outside of the previous three regions). The annotation information for CpG islands was obtained from the UCSC Genome Browser website (http://genome.ucsc.edu) (Karemaker and Vermeulen 2018).

#### 2.4.3 Functional enrichment analysis

Enriched Gene Ontology (GO) terms were identified utilizing the Metascape software (http://metascape.org) (Karolchik *et al.* 2003). The gene list for functional enrichment analysis consisted of genes that contained DMRs within their promoters (within 2 kb from TSS) and/or gene bodies. For the GO enrichment analysis, only the terms associated with “biological process” were selected. P-values were adjusted for multiple comparisons using the Benjamini–Hochberg method to control the false discovery rate (FDR) (Zhou *et al.* 2019).

## 3 Results

### 3.1 Assessing the overall performance of scDMV

In the difference experiments, all 1000 regions in the simulated data are designated as DMRs, while in the indifference experiments, all 1000 regions exhibit indifferent methylation. To ensure the validity of the simulated data, we compared its distribution with that of real data (specifically, the data from chromosome 1 in scBS-seq), as depicted in Supplementary Fig. S1 of the Supplementary Appendix. The figure leads to the following observations: (i) both the simulated data and the real data exhibit an inflated range of 0–1; (ii) the distribution of the simulated data closely aligns with that of the real data.

We assess the collective performance of scDMV and contrasting algorithms (Methylpy, CGmapTools) by analyzing averaged results from five simulation experiments. The outcomes at a *P*-value cutoff of 0.01 are illustrated in Table 1, while comprehensive results from all five experiments can be found in Supplementary Tables S1–S3 of the Supplementary Appendix.

**Table 1.** Simulation results.

Method	scDMV	CGmapTools	methylpy
*P* ≤ 0.01			
Δ≥0			
Difference	864	549	999
Indifference	1	10	999
Δ≥0.1			
Difference	595	531	721
Indifference	0	8	9
Δ≥0.15			
Difference	279	221	181
Indifference	0	1	0
Δ≥0.2			
Difference	90	21	7
Indifference	0	0	0

In the simulation experiments, we evaluate the algorithms using sensitivity and precision as performance metrics. Sensitivity is calculated as the ratio of the number of regions with a *P*-value not exceeding the defined cutoff to the total number of regions in the difference experiments. Precision, on the other hand, is determined by the ratio of the number of DMRs identified in the difference experiments to the total number of DMRs identified across all experiments.

To visualize the results, we plot the outcomes of the five experiments for each method at various cutoff points, with FDR represented on the horizontal axis and sensitivity on the vertical axis. Supplementary Figure S2 of the Supplementary Appendix illustrates this graph, where the black vertical line corresponds to an FDR of 0.005, indicating statistical significance when the FDR is below this threshold.

By examining the graph, it becomes evident that the scDMV algorithm consistently exhibits higher sensitivity in controlling Type I errors, particularly when Δ (the difference in methylation level) is 0 or greater. Furthermore, scDMV demonstrates superior precision compared to the other two algorithms, maintaining high sensitivity across multiple cutoff points for Δ, specifically at 0, 0.1, and 0.15.

In addition, we present precision boxplots for the three methods, depicted in Supplementary Fig. S3 of the Supplementary Appendix. As observed in Supplementary Fig. S3A, scDMV consistently attains higher precision compared to the other two methods, regardless of the screening conditions. Notably, the precision remains above 0.98 for scDMV across all scenarios.

Apart from sensitivity and precision, researchers commonly evaluate the false positive rate (FPR) of algorithms. Supplementary Figure S3B presents the FPR box plot for the three methods, clearly demonstrating that scDMV consistently maintains a lower false positive rate compared to the other two methods. Notably, the false positive rate of methylpy is notably high, which could potentially be attributed to the experimental principles underlying this method.

Based on the simulation results, the following conclusions can be drawn: scDMV demonstrates exceptional precision and sensitivity hen identifying DMRs in single-cell data. In summary, the aforementioned simulation results indicate that the scDMV method surpasses the other two methods in accurately detecting DNA methylation differences in single-cell bisulfite sequencing data.

### 3.2 scDMV exhibiting superior precision in the analysis of real data

In evaluating the performance of scDMV, we utilize precision as the evaluation criterion. Precision is defined as the proportion of correctly detected DMRs among all the identified DMRs. In the aforementioned experiments, we assume that the DMRs detected in the between-group experiments are all correctly identified, while the DMRs detected in the within-group experiments are all misidentified. As a result, we define true positives (${TP}^{\prime }$) as the number of DMRs identified in the between-group experiments (the first experiment), and false positives (${FP}^{\prime }$) as the number of DMRs found in the within-group experiments (the second experiment). Hence, precision can be calculated using the following formula:

$$P^{'}=\frac{{TP}^{'}}{{TP}^{'}+{FP}^{'}}=\frac{{TP}_{4c8c}^{'}}{{TP}_{4c8c}^{'}+{FP}_{8c8c}^{'}}$$

After data processing, the first experiment yields a total of 11 083 regions, while the second experiment results in 13 193 regions. We set *P*-value cutoffs at 0.001, 0.005, 0.01, and 0.05, along with Δ cutoffs at 0.1, 0.15, and 0.2. Consequently, we obtain experimental results for the three methods under different thresholds (refer to Supplementary Table S4 of the Supplementary Appendix for complete experimental results).

The results indicate that as the threshold becomes more stringent, the precision of both CGmapTools and scDMV methods gradually decreases, while the precision of the methylpy method remains relatively stable. Concretely, when the cutoffs of *P*-value are set to 0.001, 0.005, and 0.01 the scDMV method consistently maintains a precision level above 0.71, whereas the other two methods fail to reach a precision of 0.66. When we set *P*-value cutoff at 0.05, the precision of scDMV decreases, but it is still higher than 0.65, while the precision of two corresponding methods is lower than 0.59. When comparing the results across different thresholds, scDMV consistently exhibits higher precision compared to the other two methods. In other words, scDMV can identify a greater number of DMRs while ensuring fewer misidentified regions.

### 3.3 Characterization of regions with differential methylation

**Figure 2.**
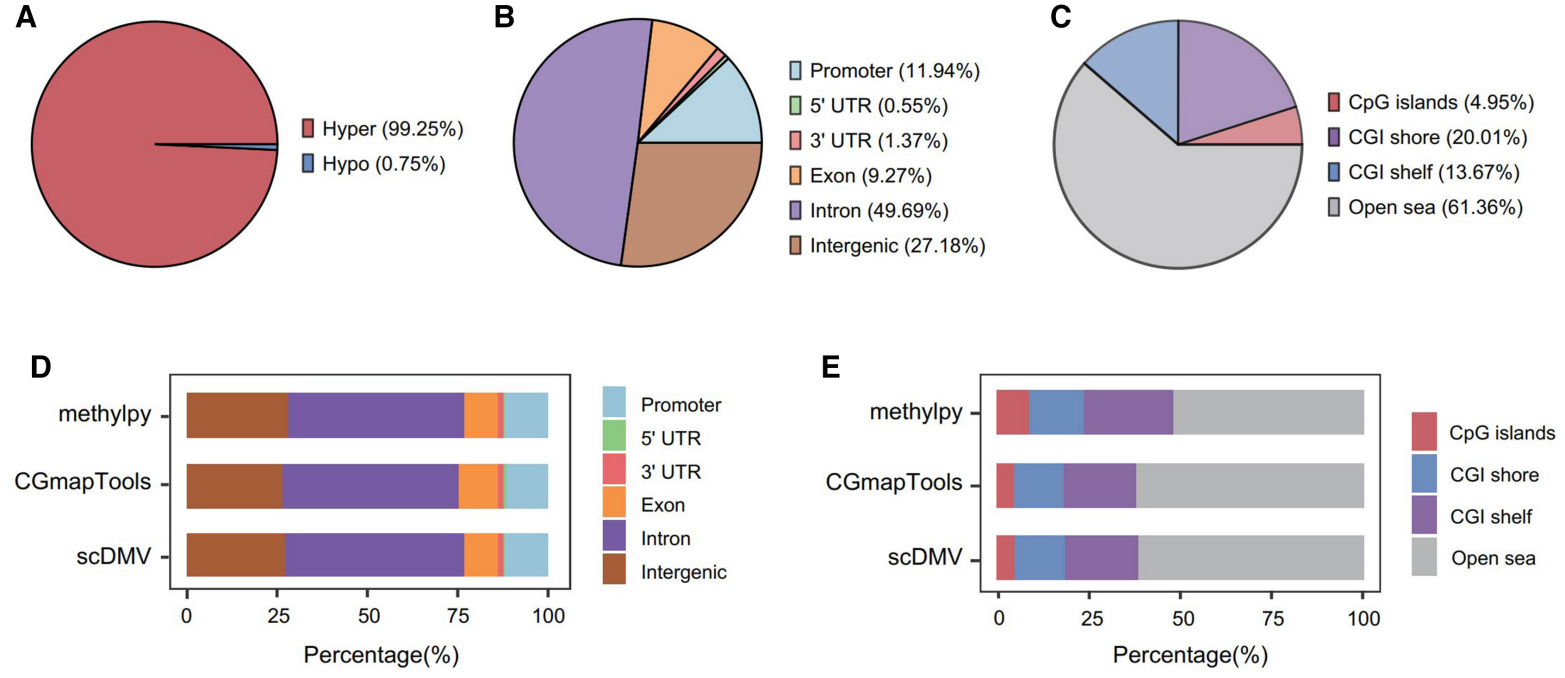
Global analysis of DMRs between 8-cell and 4-cell stages: (A) distribution of hyper-DMRs and hypo-DMRs . (B) Genomic location distribution of DMRs, including UTRs. (C) DMR distribution relative to CpG islands. (D) Genomic location fractions of DMRs identified by three methods. (E) CpG island-related location fractions of DMRs identified by three methods.

In order to accurately characterize the DMRs between the 8-cell stage and 4-cell stage, a stringent threshold was applied, selecting DMRs based on *P*-values ≤ 0.001 and Δ ≥ 0.2. As a result, a total of 1457 DMRs were identified across the entire genome. Among these DMRs, 1446 (99.25%) exhibited hypermethylation in 8-cell embryos (Fig. 2A). This observation is consistent with previous studies that have reported a significant increase in global DNA methylation levels in 8-cell embryos compared to 4-cell embryos (Benjamini and Hochberg 1995, Zhu *et al.* 2018).

To investigate the genomic distribution of DMRs, we employed the clusterProfiler package (Hanna *et al.* 2016) to annotate regions based on the human hg19 reference genome. The analysis revealed that a significant portion of the DMRs (49.69%) between consecutive developmental stages were situated within intronic regions of transcripts (Fig. 2B). In addition, 11.94% of the DMRs were identified in promoter regions, which is consistent with the association of promoter methylation with transcriptional silencing (Siegfried *et al.* 1999, Yu *et al.* 2012). It is worth noting that similar patterns of genome distribution for DMRs were observed when comparing the scDMV method with the other two methods (Fig. 2D).

Furthermore, a notable proportion (4.95%) of the DMRs detected by the scDMV tool were found to be located within CpG islands (Fig. 2C), which aligns with the genomic distribution of DMRs identified by the CGmapTools tool (Fig. 2E). In contrast, the DMRs identified by methylpy exhibited a greater preference for regions with high CpG density, such as CpG islands (9.02%) and CpG shores (14.7%) (Fig. 2E). This bias in methylpy’s DMR identification process, which involves first identifying differentially methylated sites and then merging them into DMRs, may contribute to this observation. It is possible that methods like scDMV, which directly define candidate methylated regions, achieve higher accuracy by avoiding such biases (Baylin 2005).

### 3.4 scDMV effectively capturing crucial information that is overlooked by CGmapTools

**Figure 3.**
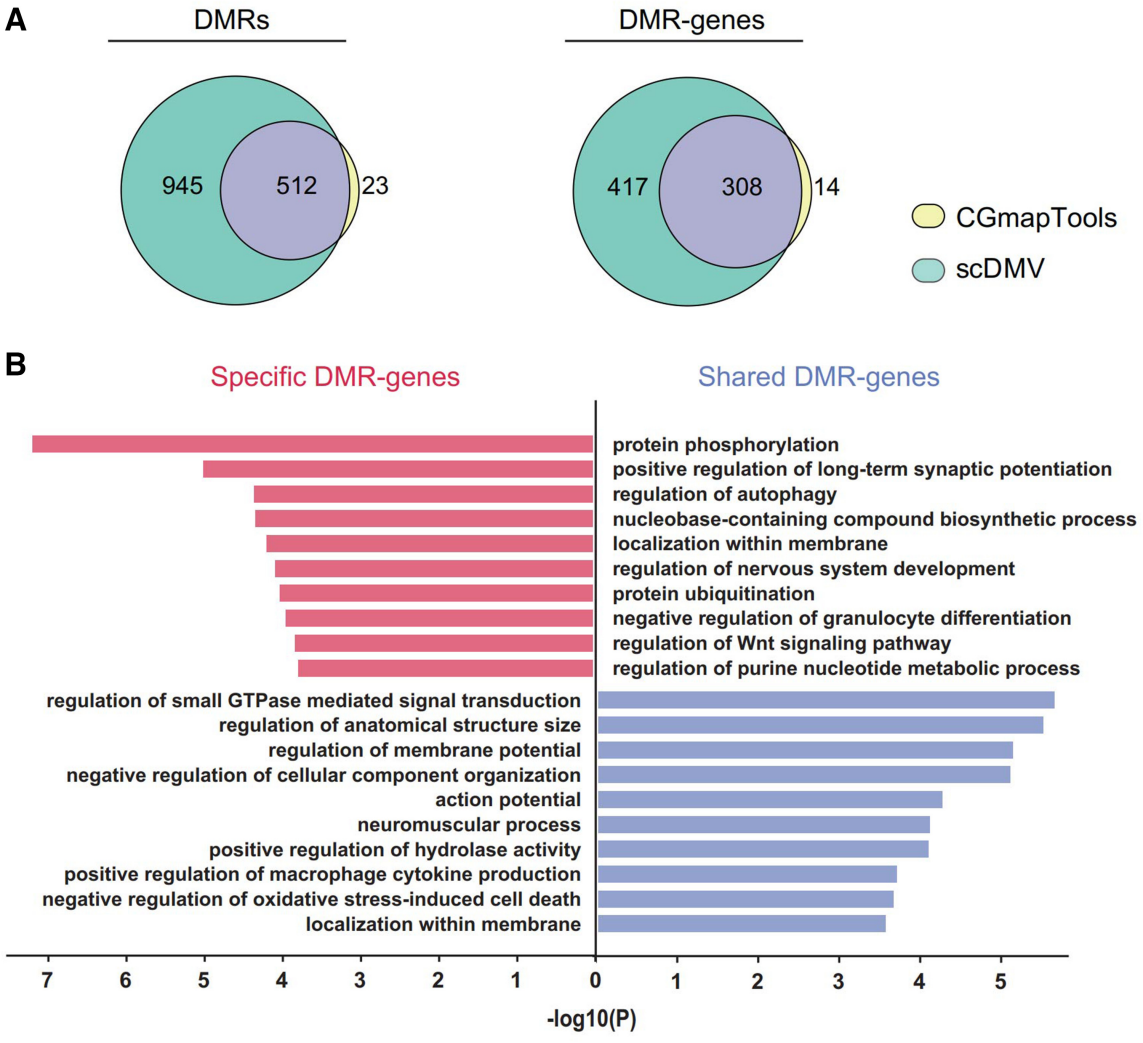
Comparison of whole-genome DMRs between single-cell WGBS methods (scDMV versus CGmapTools): (A) left: Venn diagram depicting the overlap of detected DMRs between scDMV and CGmapTools. Right: Venn diagram showing the overlap of detected DMR-genes between scDMV and CGmapTools. (B) GO enrichment analysis conducted on shared DMR-genes and specific DMR-genes identified by scDMV and CgmapTool.

Due to the high false positive rate associated with the methylpy method, we focused our comparison on the scDMV and CGmapTools methods. Among them, scDMV reported a total of 1457 DMRs, while CGmapTools reported 535 DMRs. Notably, scDMV captured 512 (95.7%) of the DMRs identified by CGmapTools, as depicted in Fig. 3A (Left). Furthermore, at the gene level, there was a substantial overlap of 308 genes between the DMRs identified by both methods, as illustrated in Fig. 3A (Right). Overall, scDMV provided a significantly greater amount of information, capturing nearly all of the DMRs reported by CGmapTools.

Given that scDMV identified a larger number of DMR-genes (417) compared to CGmapTools, it becomes intriguing to conduct functional annotation on the different DMR-genes present in the two gene lists. Through GO enrichment analysis, it was revealed that the shared DMR-genes exhibited significant enrichment in functions related to developmental regulation, such as the regulation of anatomical structure size and small GTPase-mediated signal transduction (Fig. 3B). In contrast, the specific DMR genes identified by scDMV were highly enriched in processes involving protein phosphorylation and regulation of nervous system development (Fig. 3B). Notably, previous studies have shown that phosphorylation dynamics play a dominant role in the regulated proteome during early development, and phosphorylated proteins in 8-cell embryos are associated with post-translational mechanisms (Bloom and McConnell 1990, Jühling *et al.* 2016, Peuchen *et al.* 2017). The functional enrichment of DMR-genes suggests that DNA methylation changes in genes related to protein phosphorylation may play a crucial role in embryo development, particularly during the 8-cell stage. These findings provide valuable insights into the DNA methylome dynamics between consecutive developmental stages.

To summarize, the scDMV method successfully captured nearly all of the DMRs identified by CGmapTools. In addition, scDMV revealed a broader range of significant biological events compared to CGmapTools, indicating its ability to provide more comprehensive insights.

## 4 Discussion

The scDMV method utilizes a zero–one inflated beta mixture model to detect DMRs in single-cell sequencing data, effectively handling excess zeros and ones. It demonstrates high accuracy in identifying DMRs, as shown in simulation experiments and real data analysis. The genes identified as DMRs by scDMV are involved in important functions, such as histone H3-K9 demethylation and regulation of the Wnt signaling pathway.

scDMV addresses challenges posed by low coverage and low depth in single-cell data, providing a reliable approach for DMR detection in single-cell methylation samples. Compared to existing tools, scDMV improves DMR identification accuracy. CGmapTools utilizes a dynamic fragment strategy, offering speed and low false positive rate but detecting fewer DMRs. On the other hand, the methylpy method lacks flexibility in *P*-value cutoffs and requires time-consuming multiple experiments.

scDMV combines the strengths of CGmapTools and methylpy, employing a similar dynamic fragmentation strategy and enabling DMR filtering based on user-defined thresholds. It achieves higher precision and detects more DMRs. However, the region division process in scDMV requires improvement in terms of running speed.

## Supplementary Material

btad772_Supplementary_Data
