# Supplementary material for: scDMV: a zero–one inflated beta mixture model for DNA methylation variability with scBS-seq data
Source: Bioinformatics. 2023 Dec 23;40(1):btad772. doi: 10.1093/bioinformatics/btad772 (PMC10786675; doi:10.1093/bioinformatics/btad772)
Supplement: btad772_Supplementary_Data [file btad772_supplementary_data.zip › scDMV_supplementary.docx]

**Supplementary materials of**

scDMV: A zero-one inflated beta mixture modeling strategy for differential methylation analysis with single-cell bisulfite sequencing data

Yan Zhou^1†^, Yin Zhang^1^, Minjiao Peng^2†^, Yaru Zhang^3^, Chenghao Li^3^, Lianjie Shu^4^, Yaohua Hu^1*^, Jianzhong Su^3*^ and Jinfeng Xu^5*^

^1^School of Mathematical Sciences, Institute of Statistical Sciences, Shenzhen Key Laboratory of Advanced Machine Learning and Applications, Shenzhen University, Shenzhen, China; ^2^School of Mathematics and Statistics and KLAS, Northeast Normal University, Changchun, China; ^3^School of Biomedical Engineering, School of Ophthalmology & Optometry and Eye Hospital, Wenzhou Medical University, Wenzhou, Zhejiang, China; ^4^Faculty of business administration, University of Macau; ^5^Department of Biostatistics, City University of Hong Kong.

* To whom correspondence should be addressed.

† These authors contributed to this work equally.

## Algorithm derivation

The algorithm for estimating the four-parameter model which is a random effects model with random coefficient $p_{gij}, i=1,\ldots,n_{g}$ unobserved.

Fix $\left. g \right.$and $j$, the complete likelihood takes the form

$L=\prod_{i=1}^{n_{g}} \binom{n_{gij}}{x_{gij}}p_{gij}^{x_{gij}}\left( 1-p_{gij} \right)^{n_{gij}-x_{gij}}f(p_{gij})$*,*

$f\left( p_{gij} \right)=I\left( p_{gij}=0 \right)\pi_{gj0}I\left( p_{gij}=1 \right)\pi_{gj1}I\left( 0<p_{gij}<1 \right)[\left( 1-\pi_{gj0}-\pi_{gj1} \right)Beta\left( \alpha_{gj},\beta_{gj} \right)$,

and

$\mathcal{l=}\log L=\sum_{i=1}^{n_{g}} \left[ I\left( p_{gij}=0 \right)\log\pi_{gj0}+I\left( p_{gij}=1 \right)\log\pi_{gj1}+I\left( 0<p_{gij}<1 \right)\left[ \log\left( 1-\pi_{gj0}-\pi_{gj1} \right)-\log B\left( \alpha_{gj},\beta_{gj} \right)+\log\binom{n_{gij}}{x_{gij}}+\left( x_{gij}+\alpha_{gj} \right)\log p_{gij}+\left( n_{gij}-x_{gij}+\beta_{gj} \right)\log\left( 1-p_{gij} \right) \right] \right]$.

The observed likelihood, instead, takes the form

$L_{1}=\prod_{i=1}^{n_{g}} \binom{n_{gij}}{x_{gij}}\int p_{gij}^{x_{gij}}\left( 1-p_{gij} \right)^{n_{gij}-x_{gij}}dF\left( p_{gij} \right)$,

which is difficult to compute.

Given an initial value of $\pi_{gj0}^{(0)}$, $\pi_{gj1}^{(0)}$, $\alpha_{gj}^{(0)}$, $\beta_{gj}^{(0)}$, the algorithm consists of the E-step and M-step as follows:

for $i=1,\ldots,$

(E-step), calculate

$\hat{d}_{i1}=E\left( I\left( p_{gij}=0 \right) | data,\pi_{gj0}^{\left( i-1 \right)},\pi_{gj1}^{\left( i-1 \right)},\alpha_{gj}^{\left( i-1 \right)},\beta_{gj}^{\left( i-1 \right)} \right)$,

$\hat{d}_{i2}=E\left( I\left( p_{gij}=1 \right) | data,\pi_{gj0}^{\left( i-1 \right)},\pi_{gj1}^{\left( i-1 \right)},\alpha_{gj}^{\left( i-1 \right)},\beta_{gj}^{\left( i-1 \right)} \right)$,

$\hat{d}_{i3}=E\left( I\left( 0<p_{gij}<1 \right)\log p_{gij} | data,\pi_{gj0}^{\left( i-1 \right)},\pi_{gj1}^{\left( i-1 \right)},\alpha_{gj}^{\left( i-1 \right)},\beta_{gj}^{\left( i-1 \right)} \right)$,

and

$\hat{d}_{i4}=E\left( I\left( 0<p_{gij}<1 \right)\log\left( 1-p_{gij} \right) | data,\pi_{gj0}^{\left( i-1 \right)},\pi_{gj1}^{\left( i-1 \right)},\alpha_{gj}^{\left( i-1 \right)},\beta_{gj}^{\left( i-1 \right)} \right)$,

By calculation

$\hat{d}_{i1}=I\left( x_{gij}=0 \right)\frac{\pi_{gj0}^{\left( i-1 \right)}}{\pi_{gj0}^{\left( i-1 \right)}+\left( 1-\pi_{gj0}^{\left( i-1 \right)}-\pi_{gj1}^{\left( i-1 \right)} \right)\int_{0}^{1} \frac{1}{B\left( \alpha_{gj}^{\left( i-1 \right)},\beta_{gj}^{\left( i-1 \right)} \right)}u^{\alpha_{gj}^{\left( i-1 \right)}-1}\left( 1-u \right)^{\beta_{gj}^{\left( i-1 \right)}-1}du}$

$=I\left( x_{gij}=0 \right)\frac{\pi_{gj0}^{(i-1)}}{\pi_{gj0}^{(i-1)}+\left( 1-\pi_{gj0}^{\left( i-1 \right)}-\pi_{gj1}^{(i-1)} \right)\frac{B\left( \alpha_{gj}^{(i-1)},{n_{gij}+\beta}_{gj}^{(i-1)} \right)}{B\left( \alpha_{gj}^{(i-1)},\beta_{gj}^{(i-1)} \right)}}$,

$\hat{d}_{i2}=I\left( x_{gij}=n_{gij} \right)\frac{\pi_{gj1}^{\left( i-1 \right)}}{\pi_{gj1}^{\left( i-1 \right)}+\left( 1-\pi_{gj0}^{\left( i-1 \right)}-\pi_{gj1}^{\left( i-1 \right)} \right)\int_{0}^{1} \frac{1}{B\left( \alpha_{gj}^{\left( i-1 \right)},\beta_{gj}^{\left( i-1 \right)} \right)}u^{n_{gij}+\alpha_{gj}^{\left( i-1 \right)}-1}\left( 1-u \right)^{\beta_{gj}^{\left( i-1 \right)}-1}du}$

$=I\left( x_{gij}=n_{gij} \right)\frac{\pi_{gj1}^{(i-1)}}{\pi_{gj1}^{(i-1)}+\left( 1-\pi_{gj0}^{\left( i-1 \right)}-\pi_{gj1}^{(i-1)} \right)\frac{B\left( n_{gij}+\alpha_{gj}^{(i-1)},\beta_{gj}^{(i-1)} \right)}{B\left( \alpha_{gj}^{(i-1)},\beta_{gj}^{(i-1)} \right)}}$,

$\hat{d}_{i3}=I\left( x_{gij}=0 \right)\frac{\left( 1-\pi_{gj0}^{\left( i-1 \right)}-\pi_{gj1}^{\left( i-1 \right)} \right)\int_{0}^{1} \frac{1}{B\left( \alpha_{gj}^{\left( i-1 \right)},\beta_{gj}^{\left( i-1 \right)} \right)}\left( \log u \right)u^{\alpha_{gj}^{\left( i-1 \right)}-1}\left( 1-u \right)^{{n_{gij}+\beta}_{gj}^{\left( i-1 \right)}-1}du}{\pi_{gj0}^{\left( i-1 \right)}+\left( 1-\pi_{gj0}^{\left( i-1 \right)}-\pi_{gj1}^{\left( i-1 \right)} \right)\int_{0}^{1} \frac{1}{B\left( \alpha_{gj}^{\left( i-1 \right)},\beta_{gj}^{\left( i-1 \right)} \right)}u^{\alpha_{gj}^{\left( i-1 \right)}-1}\left( 1-u \right)^{{n_{gij}+\beta}_{gj}^{\left( i-1 \right)}-1}du}$

$+I\left( x_{gij}=n_{gij} \right)\frac{\left( 1-\pi_{gj0}^{\left( i-1 \right)}-\pi_{gj1}^{\left( i-1 \right)} \right)\int_{0}^{1} \frac{1}{B\left( \alpha_{gj}^{\left( i-1 \right)},\beta_{gj}^{\left( i-1 \right)} \right)}\left( \log u \right)u^{n_{gij}+\alpha_{gj}^{\left( i-1 \right)}-1}\left( 1-u \right)^{\beta_{gj}^{\left( i-1 \right)}-1}du}{\pi_{gj1}^{\left( i-1 \right)}+\left( 1-\pi_{gj0}^{\left( i-1 \right)}-\pi_{gj1}^{\left( i-1 \right)} \right)\int_{0}^{1} \frac{1}{B\left( \alpha_{gj}^{\left( i-1 \right)},\beta_{gj}^{\left( i-1 \right)} \right)}u^{{n_{gij}+\alpha}_{gj}^{\left( i-1 \right)}-1}\left( 1-u \right)^{\beta_{gj}^{\left( i-1 \right)}-1}du}$

$+I\left( 0<x_{gij}<n_{gij} \right)\frac{\int_{0}^{1} \frac{1}{B\left( \alpha_{gj}^{\left( i-1 \right)},\beta_{gj}^{\left( i-1 \right)} \right)}\left( \log u \right)u^{x_{gij}+\alpha_{gj}^{\left( i-1 \right)}-1}\left( 1-u \right)^{n_{gij}-x_{gij}+\beta_{gj}^{\left( i-1 \right)}-1}du}{\int_{0}^{1} \frac{1}{B\left( \alpha_{gj}^{\left( i-1 \right)},\beta_{gj}^{\left( i-1 \right)} \right)}u^{x_{gij}+\alpha_{gj}^{\left( i-1 \right)}-1}\left( 1-u \right)^{n_{gij}-x_{gij}+\beta_{gj}^{\left( i-1 \right)}-1}du}$

$=I\left( x_{gij}=0 \right)\frac{\left( 1-\pi_{gj0}^{\left( i-1 \right)}-\pi_{gj1}^{(i-1)} \right)\frac{B\left( \alpha_{gj}^{(i-1)},n_{gij}+\beta_{gj}^{(i-1)} \right)}{B\left( \alpha_{gj}^{(i-1)},\beta_{gj}^{(i-1)} \right)}\left( \Psi\left( \alpha_{gj}^{(i-1)} \right)-\Psi\left( n_{gij}+\beta_{gj}^{(i-1)}+\alpha_{gj}^{(i-1)} \right) \right)}{\pi_{gj0}^{(i-1)}+\left( 1-\pi_{gj0}^{\left( i-1 \right)}-\pi_{gj1}^{(i-1)} \right)\frac{B\left( \alpha_{gj}^{(i-1)},n_{gij}+\beta_{gj}^{(i-1)} \right)}{B\left( \alpha_{gj}^{(i-1)},\beta_{gj}^{(i-1)} \right)}}$

$+I\left( x_{gij}=n_{gij} \right)\frac{\left( 1-\pi_{gj0}^{\left( i-1 \right)}-\pi_{gj1}^{(i-1)} \right)\frac{B\left( n_{gij}+\alpha_{gj}^{(i-1)},\beta_{gj}^{(i-1)} \right)}{B\left( \alpha_{gj}^{(i-1)},\beta_{gj}^{(i-1)} \right)}\left( \Psi\left( n_{gij}+\alpha_{gj}^{(i-1)} \right)-\Psi\left( n_{gij}+\beta_{gj}^{(i-1)}+\alpha_{gj}^{(i-1)} \right) \right)}{\pi_{gj1}^{(i-1)}+\left( 1-\pi_{gj0}^{\left( i-1 \right)}-\pi_{gj1}^{(i-1)} \right)\frac{B\left( n_{gij}+\alpha_{gj}^{(i-1)},\beta_{gj}^{(i-1)} \right)}{B\left( \alpha_{gj}^{(i-1)},\beta_{gj}^{(i-1)} \right)}}$

$+I\left( 0<x_{gij}<n_{gij} \right)\left( \Psi\left( x_{gij}+\alpha_{gj}^{(i-1)} \right)-\Psi\left( n_{gij}+\beta_{gj}^{(i-1)}+\alpha_{gj}^{(i-1)} \right) \right)$,

and

$\hat{d}_{i4}=I\left( x_{gij}=0 \right)\frac{\left( 1-\pi_{gj0}^{\left( i-1 \right)}-\pi_{gj1}^{(i-1)} \right)\int_{0}^{1} \frac{1}{B\left( \alpha_{gj}^{(i-1)},\beta_{gj}^{(i-1)} \right)}\log(1-u)u^{\alpha_{gj}^{(i-1)}-1}{(1-u)}^{{n_{gij}+\beta}_{gj}^{(i-1)}-1}du}{\pi_{gj0}^{(i-1)}+\left( 1-\pi_{gj0}^{\left( i-1 \right)}-\pi_{gj1}^{(i-1)} \right)\int_{0}^{1} \frac{1}{B\left( \alpha_{gj}^{(i-1)},\beta_{gj}^{(i-1)} \right)}u^{\alpha_{gj}^{(i-1)}-1}{(1-u)}^{{n_{gij}+\beta}_{gj}^{(i-1)}-1}du}$

$+I\left( x_{gij}=n_{gij} \right)\frac{\left( 1-\pi_{gj0}^{\left( i-1 \right)}-\pi_{gj1}^{(i-1)} \right)\int_{0}^{1} \frac{1}{B\left( \alpha_{gj}^{(i-1)},\beta_{gj}^{(i-1)} \right)}\log(1-u)u^{n_{gij}+\alpha_{gj}^{(i-1)}-1}{(1-u)}^{\beta_{gj}^{(i-1)}-1}du}{\pi_{gj1}^{(i-1)}+\left( 1-\pi_{gj0}^{\left( i-1 \right)}-\pi_{gj1}^{(i-1)} \right)\int_{0}^{1} \frac{1}{B\left( \alpha_{gj}^{(i-1)},\beta_{gj}^{(i-1)} \right)}u^{{n_{gij}+\alpha}_{gj}^{(i-1)}-1}{(1-u)}^{\beta_{gj}^{(i-1)}-1}du}$

$+I\left( 0<x_{gij}<n_{gij} \right)\frac{\int_{0}^{1} \frac{1}{B\left( \alpha_{gj}^{(i-1)},\beta_{gj}^{(i-1)} \right)}\log(1-u)u^{x_{gij}+\alpha_{gj}^{(i-1)}-1}{(1-u)}^{n_{gij}-x_{gij}+\beta_{gj}^{(i-1)}-1}du}{\int_{0}^{1} \frac{1}{B\left( \alpha_{gj}^{(i-1)},\beta_{gj}^{(i-1)} \right)}u^{x_{gij}+\alpha_{gj}^{(i-1)}-1}{(1-u)}^{n_{gij}-x_{gij}+\beta_{gj}^{(i-1)}-1}du}$

$=I\left( x_{gij}=0 \right)\frac{\left( 1-\pi_{gj0}^{\left( i-1 \right)}-\pi_{gj1}^{(i-1)} \right)\frac{B\left( \alpha_{gj}^{(i-1)},n_{gij}+\beta_{gj}^{(i-1)} \right)}{B\left( \alpha_{gj}^{(i-1)},\beta_{gj}^{(i-1)} \right)}\left( \Psi\left( \beta_{gj}^{(i-1)}+n_{gij} \right)-\Psi\left( n_{gij}+\beta_{gj}^{(i-1)}+\alpha_{gj}^{(i-1)} \right) \right)}{\pi_{gj0}^{(i-1)}+\left( 1-\pi_{gj0}^{\left( i-1 \right)}-\pi_{gj1}^{(i-1)} \right)\frac{B\left( \alpha_{gj}^{(i-1)},n_{gij}+\beta_{gj}^{(i-1)} \right)}{B\left( \alpha_{gj}^{(i-1)},\beta_{gj}^{(i-1)} \right)}}$

$+I\left( x_{gij}=n_{gij} \right)\frac{\left( 1-\pi_{gj0}^{\left( i-1 \right)}-\pi_{gj1}^{(i-1)} \right)\frac{B\left( n_{gij}+\alpha_{gj}^{(i-1)},\beta_{gj}^{(i-1)} \right)}{B\left( \alpha_{gj}^{(i-1)},\beta_{gj}^{(i-1)} \right)}\left( \Psi\left( \beta_{gj}^{(i-1)} \right)-\Psi\left( n_{gij}+\beta_{gj}^{(i-1)}+\alpha_{gj}^{(i-1)} \right) \right)}{\pi_{gj1}^{(i-1)}+\left( 1-\pi_{gj0}^{\left( i-1 \right)}-\pi_{gj1}^{(i-1)} \right)\frac{B\left( n_{gij}+\alpha_{gj}^{(i-1)},\beta_{gj}^{(i-1)} \right)}{B\left( \alpha_{gj}^{(i-1)},\beta_{gj}^{(i-1)} \right)}}$

$+I\left( 0<x_{gij}<n_{gij} \right)\left( \Psi\left( n_{gij}-x_{gij}+\beta_{gj}^{(i-1)} \right)-\Psi\left( n_{gij}+\beta_{gj}^{(i-1)}+\alpha_{gj}^{(i-1)} \right) \right)$.

In the M step, maximize the objective function to update the estimates to $\pi_{gj0}^{(i)}$, $\pi_{gj1}^{(i)}$, $\alpha_{gj}^{(i)}$, $\beta_{gj}^{(i)}$:

${\mathcal{l=}L}\left( \pi_{gj0},\pi_{gj1},\alpha_{gj},\beta_{gj} \right)=\sum_{i=1}^{n_{g}} \left\{ \hat{d}_{i1}\log\pi_{gj0}+\hat{d}_{i2}\log\pi_{gj1}+\left( 1-\hat{d}_{i1}-\hat{d}_{i2} \right)\left[ \log\left( 1-\pi_{gj0}-\pi_{gj1} \right)+\log\binom{n_{gij}}{x_{gij}}-\log B\left( \alpha_{gj},\beta_{gj} \right) \right]+\left( x_{gij}+\alpha_{gj} \right)\hat{d}_{i3}+\left( n_{gij}-x_{gij}+\beta_{gj} \right)\hat{d}_{i4} \right\}$.

We let $\alpha_{gj}=e^{\alpha_{gj}^{'}}$, $\beta_{gj}=e^{\beta_{gj}^{'}}$, and then we can get:

${L_{1}}^{'}\left( \pi_{gj0},\pi_{gj1},\alpha_{gj}^{'},\beta_{gj}^{'} \right)=\sum_{i=1}^{n_{g}} \left\{ \hat{d}_{i1}\log\pi_{gj0}+\hat{d}_{i2}\log\pi_{gj1}+\left( 1-\hat{d}_{i1}-\hat{d}_{i2} \right)\left[ \log\left( 1-\pi_{gj0}-\pi_{gj1} \right)+\log\binom{n_{gij}}{x_{gij}}-\log B\left( e^{\alpha_{gj}^{'}},e^{\beta_{gj}^{'}} \right) \right]+\left( x_{gij}+e^{\alpha_{gj}^{'}}, \right)\hat{d}_{i3}+\left( n_{gij}-x_{gij}+e^{\beta_{gj}^{'}} \right)\hat{d}_{i4} \right\}$.

We use maximum likelihood estimation to estimate parameters:

$\frac{\partial{L_{1}}^{'}}{\partial\pi_{gj0}}=\sum_{i=1}^{n_{g}} \left( \frac{\hat{d}_{i1}}{\pi_{gj0}}-\frac{1-\hat{d}_{i1}-\hat{d}_{i2}}{1-\pi_{gj0}-\pi_{gj1}} \right)=0$,

$\frac{\partial{L_{1}}^{'}}{\partial\pi_{gj1}}=\sum_{i=1}^{n_{g}} \left( \frac{\hat{d}_{i2}}{\pi_{gj1}}-\frac{1-\hat{d}_{i1}-\hat{d}_{i2}}{1-\pi_{gj0}-\pi_{gj1}} \right)=0$,

$\frac{\partial{L_{1}}^{'}}{\partial\alpha_{gj1}^{'}}=\sum_{i=1}^{n_{g}} \left( -\left( 1-\hat{d}_{i1}-\hat{d}_{i2} \right)e^{\alpha_{gj}^{'}}\left( \Psi\left( e^{\alpha_{gj}^{'}} \right)-\Psi\left( e^{\alpha_{gj}^{'}}+e^{\beta_{gj}^{'}} \right) \right)+\hat{d}_{i3}e^{\alpha_{gj}^{'}} \right)=0$,

$\frac{\partial{L_{1}}^{'}}{\partial\beta_{gj1}^{'}}=\sum_{i=1}^{n_{g}} \left( -\left( 1-\hat{d}_{i1}-\hat{d}_{i2} \right)e^{\beta_{gj}^{'}}\left( \Psi\left( e^{\beta_{gj}^{'}} \right)-\Psi\left( e^{\alpha_{gj}^{'}}+e^{\beta_{gj}^{'}} \right) \right)+\hat{d}_{i4}e^{\beta_{gj}^{'}} \right)=0$.

We can solve that: $\pi_{gj0}=\frac{\sum_{i=1}^{n_{g}} \hat{d}_{i1}}{n_{g}}$, $\pi_{gj1}=\frac{\sum_{i=1}^{n_{g}} \hat{d}_{i2}}{n_{g}}$ and

$\Psi\left( e^{\alpha_{gj}^{'}} \right)-\Psi\left( e^{\alpha_{gj}^{'}}+e^{\beta_{gj}^{'}} \right)=\frac{\sum_{i=1}^{n_{g}} \hat{d}_{i3}}{\sum_{i=1}^{n_{g}} (1-\hat{d}_{i1}-\hat{d}_{i2})}$,

$\Psi\left( e^{\beta_{gj}^{'}} \right)-\Psi\left( e^{\alpha_{gj}^{'}}+e^{\beta_{gj}^{'}} \right)=\frac{\sum_{i=1}^{n_{g}} \hat{d}_{i4}}{\sum_{i=1}^{n_{g}} (1-\hat{d}_{i1}-\hat{d}_{i2})}$.

Let $C_{1}=\frac{\sum_{i=1}^{n_{g}} \hat{d}_{i3}}{\sum_{i=1}^{n_{g}} (1-\hat{d}_{i1}-\hat{d}_{i2})}$, $C_{2}=\frac{\sum_{i=1}^{n_{g}} \hat{d}_{i4}}{\sum_{i=1}^{n_{g}} (1-\hat{d}_{i1}-\hat{d}_{i2})}$. We use Newton method to solve $\alpha_{gj}^{'}$ and $\beta_{gj}^{'}$:

We construct function $f_{1}$ and $f_{2}$:

$f_{1}\left( \alpha_{gj}^{'},\beta_{gj}^{'} \right)=\Psi\left( e^{\alpha_{gj}^{'}} \right)-\Psi\left( e^{\alpha_{gj}^{'}}+e^{\beta_{gj}^{'}} \right)-C_{1}$,

$f_{2}\left( \alpha_{gj}^{'},\beta_{gj}^{'} \right)=\Psi\left( e^{\beta_{gj}^{'}} \right)-\Psi\left( e^{\alpha_{gj}^{'}}+e^{\beta_{gj}^{'}} \right)-C_{2}$.

And then we can get *Jac* matrix:

$Jac=\left( \begin{matrix} \frac{\partial f_{1}}{\partial\alpha_{gj}^{'}} & \frac{\partial f_{1}}{\partial\beta_{gj}^{'}} \\ \frac{\partial f_{2}}{\partial\alpha_{gj}^{'}} & \frac{\partial f_{2}}{\partial\beta_{gj}^{'}} \end{matrix} \right)$.

Calculate the elements of the matrix:

$\frac{\partial f_{1}}{\partial\alpha_{gj}^{'}}=e^{\alpha_{gj}^{'}}\left( \Psi^{'}\left( e^{\alpha_{gj}^{'}} \right)-\Psi^{'}\left( e^{\alpha_{gj}^{'}}+e^{\beta_{gj}^{'}} \right) \right)$,

$\frac{\partial f_{1}}{\partial\beta_{gj}^{'}}={-e}^{\beta_{gj}^{'}}\left. \Psi^{'}\left( e^{\alpha_{gj}^{'}}+e^{\beta_{gj}^{'}} \right) \right.$,

$\frac{\partial f_{2}}{\partial\alpha_{gj}^{'}}={-e}^{\alpha_{gj}^{'}}\left. \Psi^{'}\left( e^{\alpha_{gj}^{'}}+e^{\beta_{gj}^{'}} \right) \right.$,

$\frac{\partial f_{2}}{\partial\beta_{gj}^{'}}=e^{\beta_{gj}^{'}}\left( \Psi^{'}\left( e^{\beta_{gj}^{'}} \right)-\Psi^{'}\left( e^{\alpha_{gj}^{'}}+e^{\beta_{gj}^{'}} \right) \right)$.

Finally, the matrix is expressed as:

$Jac=\left( \begin{matrix} e^{\alpha_{gj}^{'}}\left( \Psi^{'}\left( e^{\alpha_{gj}^{'}} \right)-\Psi^{'}\left( e^{\alpha_{gj}^{'}}+e^{\beta_{gj}^{'}} \right) \right) & {-e}^{\beta_{gj}^{'}}\left. \Psi^{'}\left( e^{\alpha_{gj}^{'}}+e^{\beta_{gj}^{'}} \right) \right. \\ {-e}^{\alpha_{gj}^{'}}\left. \Psi^{'}\left( e^{\alpha_{gj}^{'}}+e^{\beta_{gj}^{'}} \right) \right. & e^{\beta_{gj}^{'}}\left( \Psi^{'}\left( e^{\beta_{gj}^{'}} \right)-\Psi^{'}\left( e^{\alpha_{gj}^{'}}+e^{\beta_{gj}^{'}} \right) \right) \end{matrix} \right)$.

And then we can calculate $\alpha_{gj}^{'}$ and $\beta_{gj}^{'}$:

$\binom{\alpha_{gj}^{'(i)}}{\beta_{gj}^{'(i)}}=\binom{\alpha_{gj}^{'(i-1)}}{\beta_{gj}^{'(i-1)}}-{Jac}^{-1}\binom{f_{1}\left( \alpha_{gj}^{'(i-1)},\beta_{gj}^{'(i-1)} \right)}{f_{2}\left( \alpha_{gj}^{'(i-1)},\beta_{gj}^{'(i-1)} \right)}$.

We use $\alpha_{gj}=e^{\alpha_{gj}^{'}}$, $\beta_{gj}=e^{\beta_{gj}^{'}}$ to estimate $\alpha_{gj}$ and $\beta_{gj}$.

We obtain the maximum likelihood by estimating $\hat{\pi}_{gj0}^{(i)}$, $\hat{\pi}_{gj1}^{(i)}$, $\hat{\alpha}_{gj}^{(i)}$, $\hat{\beta}_{gj}^{(i)}$ upon the convergence and the maximized log likelihood function value is then$\mathcal{l}\left( \hat{\pi}_{gj0,}\hat{\pi}_{gj1},\hat{\alpha}_{gj},\hat{\beta}_{gj} | data,\hat{\pi}_{gj0,}\hat{\pi}_{gj1},\hat{\alpha}_{gj},\hat{\beta}_{gj} \right)$. Based on this, LRT test statistic can be obtained for our proposed test statistic.

We test it by Wald test method:

$p_{gij}=\left\{ \begin{aligned} \pi_{gj0} p_{gij}=0 \\ \pi_{gj1} p_{gij}=1 \\ \left( 1-\pi_{gj0}-\pi_{gj1} \right)Beta\left( \alpha_{gj},\beta_{gj} \right) p_{gij}\epsilon(0,1) \end{aligned} \right.$.

We take the logarithmic likelihood first derivative of $L$, and we get:

$\frac{\partial\log L}{\partial\pi_{gj0}}=\frac{I(p_{gij}=0)}{\pi_{gj0}}-\frac{I(0<p_{gij}<1)}{1-\pi_{gj0}-\pi_{gj1}}$,

$\frac{\partial\log L}{\partial\pi_{gj1}}=\frac{I(p_{gij}=1)}{\pi_{gj1}}-\frac{I(0<p_{gij}<1)}{1-\pi_{gj0}-\pi_{gj1}}$,

$\frac{\partial\log L}{\partial\alpha_{gj}}=I\left( 0<p_{gij}<1 \right)\log p_{gij}-I(0<p_{gij}<1)\frac{\frac{\partial B\left( \alpha_{gj},\beta_{gj} \right)}{\partial\alpha_{gj}}}{B\left( \alpha_{gj},\beta_{gj} \right)}$,

$\frac{\partial\log L}{\partial\beta_{gj}}=I\left( 0<p_{gij}<1 \right)\log(1-p_{gij})-I(0<p_{gij}<1)\frac{\frac{\partial B\left( \alpha_{gj},\beta_{gj} \right)}{\partial\beta_{gj}}}{B\left( \alpha_{gj},\beta_{gj} \right)}$.

And then we take the logarithmic likelihood second derivative of $L$ to get that:

$\frac{\partial^{2}\log L}{\partial\pi_{gj0}^{2}}=-\frac{I(p_{gij}=0)}{\pi_{gj0}^{2}}-\frac{I(0<p_{gij}<1)}{{(1-\pi_{gj0}-\pi_{gj1})}^{2}}$,

$\frac{\partial^{2}\log L}{\partial\pi_{gj0}\partial\pi_{gj1}}=-\frac{I(0<p_{gij}<1)}{{(1-\pi_{gj0}-\pi_{gj1})}^{2}}$,

$\frac{\partial^{2}\log L}{\partial\pi_{gj1}^{2}}=-\frac{I(p_{gij}=1)}{\pi_{gj1}^{2}}-\frac{I(0<p_{gij}<1)}{{(1-\pi_{gj0}-\pi_{gj1})}^{2}}$,

$\frac{\partial^{2}\log L}{\partial\pi_{gj1}\partial\pi_{gj0}}=-\frac{I(0<p_{gij}<1)}{{(1-\pi_{gj0}-\pi_{gj1})}^{2}}$,

$\frac{\partial^{2}\log L}{\partial\pi_{gj0}\partial\alpha_{gj}}=\frac{\partial^{2}\log L}{\partial\pi_{gj0}\partial\beta_{gj}}=\frac{\partial^{2}\log L}{\partial\pi_{gj1}\partial\alpha_{gj}}=\frac{\partial^{2}\log L}{\partial\pi_{gj1}\partial\beta_{gj}}=0$,

$\frac{\partial^{2}\log L}{\partial\alpha_{gj}^{2}}=-I(0<p_{gij}<1)\frac{\frac{\partial^{2}B\left( \alpha_{gj},\beta_{gj} \right)}{\partial\alpha_{gj}^{2}}-\left( \frac{\partial B\left( \alpha_{gj},\beta_{gj} \right)}{\partial\alpha_{gj}} \right)^{2}}{B^{2}\left( \alpha_{gj},\beta_{gj} \right)}$,

$\frac{\partial^{2}\log L}{\partial\beta_{gj}^{2}}=-I(0<p_{gij}<1)\frac{\frac{\partial^{2}B\left( \alpha_{gj},\beta_{gj} \right)}{\partial\beta_{gj}^{2}}-\left( \frac{\partial B\left( \alpha_{gj},\beta_{gj} \right)}{\partial\beta_{gj}} \right)^{2}}{B^{2}\left( \alpha_{gj},\beta_{gj} \right)}$,

$\frac{\partial^{2}\log L}{\partial\alpha_{gj}\partial\beta_{gj}}=\frac{\partial^{2}\log L}{\partial\beta_{gj}\partial\alpha_{gj}}-I(0<p_{gij}<1)\frac{\frac{\partial^{2}B\left( \alpha_{gj},\beta_{gj} \right)}{\partial\alpha_{gj}\partial\beta_{gj}}-\left. \frac{\partial B\left( \alpha_{gj},\beta_{gj} \right)}{\partial\alpha_{gj}}\frac{\partial B\left( \alpha_{gj},\beta_{gj} \right)}{\partial\beta_{gj}} \right.}{B^{2}\left( \alpha_{gj},\beta_{gj} \right)}$,

The information matrix is:

$I\left( \pi_{gj0},\pi_{gj1},\alpha_{gj},\beta_{gj} \right)=-E\left( \begin{matrix} \begin{matrix} \frac{\partial^{2}\log L}{\partial\pi_{gj0}^{2}} & \frac{\partial^{2}\log L}{\partial\pi_{gj0}\partial\pi_{gj1}} \\ \frac{\partial^{2}\log L}{\partial\pi_{gj1}\partial\pi_{gj0}} & \frac{\partial^{2}\log L}{\partial\pi_{gj1}^{2}} \end{matrix} & \begin{matrix} 0 & 0 \\ 0 & 0 \end{matrix} \\ \begin{matrix} 0 & 0 \\ 0 & 0 \end{matrix} & \begin{matrix} \frac{\partial^{2}\log L}{\partial\alpha_{gj}^{2}} & \frac{\partial^{2}\log L}{\partial\alpha_{gj}\partial\beta_{gj}} \\ \frac{\partial^{2}\log L}{\partial\beta_{gj}\partial\alpha_{gj}} & \frac{\partial^{2}\log L}{\partial\beta_{gj}^{2}} \end{matrix} \end{matrix} \right)$,

$EI\left( p_{gij}=0 \right)=\pi_{gj0}$,

$EI\left( p_{gij}=1 \right)=\pi_{gj1}$,

$EI\left( {0<p}_{gij}<1 \right)=1-\pi_{gj0}-\pi_{gj1}$,

$E\frac{\partial^{2}\log L}{\partial\pi_{gj0}^{2}}=-\frac{1}{\pi_{gj0}}-\frac{1}{1-\pi_{gj0}-\pi_{gj1}}=-\frac{1-\pi_{gj1}}{\pi_{gj0}\left( 1-\pi_{gj0}-\pi_{gj1} \right)}$,

$E\frac{\partial^{2}\log L}{\partial\pi_{gj1}^{2}}=-\frac{1}{\pi_{gj1}}-\frac{1}{1-\pi_{gj0}-\pi_{gj1}}=-\frac{1-\pi_{gj0}}{\pi_{gj1}\left( 1-\pi_{gj0}-\pi_{gj1} \right)}$,

$E\frac{\partial^{2}\log L}{\partial\pi_{gj0}\partial\pi_{gj1}}=E\frac{\partial^{2}\log L}{\partial\pi_{gj1}\partial\pi_{gj0}}=-\frac{1}{1-\pi_{gj0}-\pi_{gj1}}$,

$E\frac{\partial^{2}\log L}{\partial\alpha_{gj}^{2}}=-\left( 1-\pi_{gj0}-\pi_{gj1} \right)\cdot$

$\left( \frac{\left( \Psi\left( \alpha_{gj} \right)-\Psi\left( \alpha_{gj}+\beta_{gj} \right) \right)^{2}+\left( \Psi^{‘}\left( \alpha_{gj} \right)-\Psi^{’}\left( \alpha_{gj}+\beta_{gj} \right) \right)-B\left( \alpha_{gj},\beta_{gj} \right)\left( \Psi\left( \alpha_{gj} \right)-\Psi\left( \alpha_{gj}+\beta_{gj} \right) \right)^{2}}{B\left( \alpha_{gj},\beta_{gj} \right)} \right)$,

$E\frac{\partial^{2}\log L}{\partial\beta_{gj}^{2}}=-\left( 1-\pi_{gj0}-\pi_{gj1} \right)\cdot$

$\left( \frac{\left( \Psi\left( \beta_{gj} \right)-\Psi\left( \alpha_{gj}+\beta_{gj} \right) \right)^{2}+\left( \Psi^{‘}\left( \beta_{gj} \right)-\Psi^{’}\left( \alpha_{gj}+\beta_{gj} \right) \right)-B\left( \alpha_{gj},\beta_{gj} \right)\left( \Psi\left( \beta_{gj} \right)-\Psi\left( \alpha_{gj}+\beta_{gj} \right) \right)^{2}}{B\left( \alpha_{gj},\beta_{gj} \right)} \right)$,

$E\frac{\partial^{2}\log L}{\partial\alpha_{gj}\partial\beta_{gj}}=E\frac{\partial^{2}\log L}{\partial\beta_{gj}\partial\alpha_{gj}}=-\left( 1-\pi_{gj0}-\pi_{gj1} \right)\cdot$

$\left( \frac{\left( \Psi\left( \alpha_{gj} \right)-\Psi\left( \alpha_{gj}+\beta_{gj} \right) \right)\left( \Psi\left( \beta_{gj} \right)-\Psi\left( \alpha_{gj}+\beta_{gj} \right) \right)-\left. \Psi^{’}\left( \alpha_{gj}+\beta_{gj} \right) \right.-B\left( \alpha_{gj},\beta_{gj} \right)\left( \Psi\left( \alpha_{gj} \right)-\Psi\left( \alpha_{gj}+\beta_{gj} \right) \right)\left( \Psi\left( \beta_{gj} \right)-\Psi\left( \alpha_{gj}+\beta_{gj} \right) \right)}{B\left( \alpha_{gj},\beta_{gj} \right)} \right).$

We can get the information matrix is:

$I\left( \pi_{gj0},\pi_{gj1},\alpha_{gj},\beta_{gj} \right)=\left( \begin{matrix} \begin{matrix} -E\frac{\partial^{2}\log L}{\partial\pi_{gj0}^{2}} & -E\frac{\partial^{2}\log L}{\partial\pi_{gj0}\partial\pi_{gj1}} \\ -E\frac{\partial^{2}\log L}{\partial\pi_{gj1}\partial\pi_{gj0}} & -E\frac{\partial^{2}\log L}{\partial\pi_{gj1}^{2}} \end{matrix} & \begin{matrix} 0 & 0 \\ 0 & 0 \end{matrix} \\ \begin{matrix} 0 & 0 \\ 0 & 0 \end{matrix} & \begin{matrix} -E\frac{\partial^{2}\log L}{\partial\alpha_{gj}^{2}} & -E\frac{\partial^{2}\log L}{\partial\alpha_{gj}\partial\beta_{gj}} \\ -E\frac{\partial^{2}\log L}{\partial\beta_{gj}\partial\alpha_{gj}} & -E\frac{\partial^{2}\log L}{\partial\beta_{gj}^{2}} \end{matrix} \end{matrix} \right)$.

We test $H_{0}:Ep_{1j}=Ep_{2j}$ against $H_{1}:Ep_{1j}\neq Ep_{2j}$

$\left. g\left( \theta_{g} \right) \right.=Ep_{gj}=\pi_{gj1}+\left( 1-\pi_{gj0}-\pi_{gj1} \right)\frac{\alpha_{gj}}{\alpha_{gj}+\beta_{gj}}$,

$\theta_{g}=\left( \pi_{gj0},\pi_{gj1}, \alpha_{gj}, \beta_{gj} \right)$ $\left. g=1,2 \right.$.

We can get the Wald statistic:

$T=\frac{g\left( \hat{\theta}_{1} \right)-g\left( \hat{\theta}_{2} \right)}{\sqrt{\left( \frac{\partial g\left( \theta_{1} \right)}{\partial\theta_{1}} \right)^{T}I\left( \theta_{1} \right)\frac{\partial g\left( \theta_{1} \right)}{\partial\theta_{1}}+\left( \frac{\partial g\left( \theta_{2} \right)}{\partial\theta_{2}} \right)^{T}I\left( \theta_{2} \right)\frac{\partial g\left( \theta_{2} \right)}{\partial\theta_{2}}}}\sim N\left( 0,1 \right)$.

**Table S1. Results of five simulation experiments of CGmapTools**

| CGmapTools | | | Simulation1 | Simulation2 | Simulation3 | Simulation4 | Simulation5 |
| --- | --- | --- | --- | --- | --- | --- | --- |
| pvalue≤0.001 | Δ≥0 | difference | 246 | 229 | 254 | 241 | 233 |
|  |  | no difference | 3 | 3 | 1 | 2 | 2 |
|  | Δ≥0.1 | difference | 243 | 228 | 254 | 241 | 233 |
|  |  | no difference | 3 | 3 | 1 | 2 | 2 |
|  | Δ≥0.15 | difference | 152 | 153 | 181 | 160 | 158 |
|  |  | no difference | 1 | 0 | 0 | 1 | 0 |
|  | Δ≥0.2 | difference | 22 | 22 | 20 | 25 | 16 |
|  |  | no difference | 0 | 0 | 0 | 0 | 0 |
| Pvalue≤0.005 | Δ≥0 | difference | 441 | 413 | 448 | 431 | 433 |
|  |  | no difference | 6 | 7 | 2 | 5 | 8 |
|  | Δ≥0.1 | difference | 434 | 406 | 439 | 425 | 430 |
|  |  | no difference | 6 | 6 | 2 | 4 | 7 |
|  | Δ≥0.15 | difference | 193 | 209 | 230 | 200 | 225 |
|  |  | no difference | 1 | 0 | 0 | 1 | 1 |
|  | Δ≥0.2 | difference | 22 | 22 | 21 | 25 | 17 |
|  |  | no difference | 0 | 0 | 0 | 0 | 0 |
| pvalue≤0.01 | Δ≥0 | difference | 560 | 529 | 550 | 547 | 561 |
|  |  | no difference | 11 | 10 | 8 | 10 | 13 |
|  | Δ≥0.1 | difference | 544 | 513 | 527 | 527 | 542 |
|  |  | no difference | 10 | 8 | 6 | 7 | 10 |
|  | Δ≥0.15 | difference | 203 | 215 | 240 | 211 | 234 |
|  |  | no difference | 1 | 0 | 0 | 1 | 1 |
|  | Δ≥0.2 | difference | 22 | 22 | 21 | 25 | 17 |
|  |  | no difference | 0 | 0 | 0 | 0 | 0 |
| pvalue≤0.05 | Δ≥0 | difference | 816 | 817 | 772 | 800 | 800 |
|  |  | no difference | 48 | 54 | 42 | 51 | 48 |
|  | Δ≥0.1 | difference | 687 | 691 | 668 | 678 | 683 |
|  |  | no difference | 18 | 21 | 16 | 16 | 18 |
|  | Δ≥0.15 | difference | 205 | 220 | 243 | 215 | 234 |
|  |  | no difference | 1 | 0 | 0 | 1 | 2 |
|  | Δ≥0.2 | difference | 22 | 22 | 21 | 25 | 17 |
|  |  | no difference | 0 | 0 | 0 | 0 | 0 |

## Table S2. Results of five simulation experiments of Methylpy

| scDMV | | | Simulation1 | Simulation2 | Simulation3 | Simulation4 | Simulation5 |
| --- | --- | --- | --- | --- | --- | --- | --- |
| pvalue≤0.001 | Δ≥0 | difference | 999 | 999 | 999 | 999 | 999 |
|  |  | no difference | 999 | 999 | 999 | 999 | 999 |
|  | Δ≥0.1 | difference | 720 | 725 | 708 | 720 | 732 |
|  |  | no difference | 11 | 9 | 5 | 9 | 9 |
|  | Δ≥0.15 | difference | 189 | 190 | 177 | 178 | 170 |
|  |  | no difference | 0 | 0 | 0 | 1 | 0 |
|  | Δ≥0.2 | difference | 14 | 4 | 4 | 10 | 5 |
|  |  | no difference | 0 | 0 | 0 | 0 | 0 |
| pvalue≤0.005 | Δ≥0 | difference | 999 | 999 | 999 | 999 | 999 |
|  |  | no difference | 999 | 999 | 999 | 999 | 999 |
|  | Δ≥0.1 | difference | 720 | 725 | 708 | 720 | 732 |
|  |  | no difference | 11 | 9 | 5 | 9 | 9 |
|  | Δ≥0.15 | difference | 189 | 190 | 177 | 178 | 170 |
|  |  | no difference | 0 | 0 | 0 | 1 | 0 |
|  | Δ≥0.2 | difference | 14 | 4 | 4 | 10 | 5 |
|  |  | no difference | 0 | 0 | 0 | 0 | 0 |
| pvalue≤0.01 | Δ≥0 | difference | 999 | 999 | 999 | 999 | 999 |
|  |  | no difference | 999 | 999 | 999 | 999 | 999 |
|  | Δ≥0.1 | difference | 720 | 725 | 708 | 720 | 732 |
|  |  | no difference | 11 | 9 | 5 | 9 | 9 |
|  | Δ≥0.15 | difference | 189 | 190 | 177 | 178 | 170 |
|  |  | no difference | 0 | 0 | 0 | 1 | 0 |
|  | Δ≥0.2 | difference | 14 | 4 | 4 | 10 | 5 |
|  |  | no difference | 0 | 0 | 0 | 0 | 0 |
| pvalue≤0.05 | Δ≥0 | difference | 999 | 999 | 999 | 999 | 999 |
|  |  | no difference | 999 | 999 | 999 | 999 | 999 |
|  | Δ≥0.1 | difference | 720 | 725 | 708 | 720 | 732 |
|  |  | no difference | 11 | 9 | 5 | 9 | 9 |
|  | Δ≥0.15 | difference | 189 | 190 | 177 | 178 | 170 |
|  |  | no difference | 0 | 0 | 0 | 1 | 0 |
|  | Δ≥0.2 | difference | 14 | 4 | 4 | 10 | 5 |
|  |  | no difference | 0 | 0 | 0 | 0 | 0 |

## Table S3. Results of five simulation experiments of scDMV

| scDMV | | | Simulation1 | Simulation2 | Simulation3 | Simulation4 | Simulation5 |
| --- | --- | --- | --- | --- | --- | --- | --- |
| pvalue≤0.001 | Δ≥0 | difference | 591 | 580 | 575 | 574 | 596 |
|  |  | no difference | 0 | 0 | 0 | 1 | 0 |
|  | Δ≥0.1 | difference | 445 | 450 | 447 | 426 | 475 |
|  |  | no difference | 0 | 0 | 0 | 1 | 0 |
|  | Δ≥0.15 | difference | 227 | 241 | 232 | 223 | 245 |
|  |  | no difference | 0 | 0 | 0 | 1 | 0 |
|  | Δ≥0.2 | difference | 67 | 87 | 77 | 74 | 72 |
|  |  | no difference | 0 | 0 | 0 | 0 | 0 |
| pvalue≤0.005 | Δ≥0 | difference | 796 | 796 | 778 | 779 | 783 |
|  |  | no difference | 0 | 1 | 0 | 1 | 0 |
|  | Δ≥0.1 | difference | 561 | 573 | 552 | 537 | 574 |
|  |  | no difference | 0 | 0 | 0 | 1 | 0 |
|  | Δ≥0.15 | difference | 259 | 278 | 266 | 268 | 278 |
|  |  | no difference | 0 | 0 | 0 | 1 | 0 |
|  | Δ≥0.2 | difference | 80 | 101 | 86 | 86 | 82 |
|  |  | no difference | 0 | 0 | 0 | 0 | 0 |
| pvalue≤0.01 | Δ≥0 | difference | 867 | 870 | 864 | 857 | 860 |
|  |  | no difference | 0 | 1 | 1 | 2 | 0 |
|  | Δ≥0.1 | difference | 599 | 603 | 586 | 577 | 610 |
|  |  | no difference | 0 | 0 | 0 | 2 | 0 |
|  | Δ≥0.15 | difference | 268 | 284 | 278 | 282 | 285 |
|  |  | no difference | 0 | 0 | 0 | 1 | 0 |
|  | Δ≥0.2 | difference | 83 | 102 | 89 | 92 | 85 |
|  |  | no difference | 0 | 0 | 0 | 0 | 0 |
| pvalue≤0.05 | Δ≥0 | difference | 971 | 976 | 965 | 964 | 963 |
|  |  | no difference | 11 | 14 | 8 | 10 | 11 |
|  | Δ≥0.1 | difference | 623 | 639 | 620 | 617 | 639 |
|  |  | no difference | 3 | 4 | 3 | 5 | 5 |
|  | Δ≥0.15 | difference | 281 | 289 | 285 | 293 | 292 |
|  |  | no difference | 1 | 0 | 1 | 2 | 3 |
|  | Δ≥0.2 | difference | 88 | 105 | 92 | 96 | 86 |
|  |  | no difference | 1 | 0 | 0 | 0 | 1 |

## Table S4. Results of three methods based on scBS-seq data

| Real Data | | | CGmapTools | Methylpy | scDMV |
| --- | --- | --- | --- | --- | --- |
| pvalu≤0.001 | Δ≥0.1 | difference | 842 | 3694 | 2742 |
|  |  | no difference | 439 | 3417 | 713 |
|  |  | precision | 0.657299 | 0.519477 | 0.793632 |
|  | Δ≥0.15 | difference | 705 | 2534 | 2102 |
|  |  | no difference | 379 | 2182 | 546 |
|  |  | precision | 0.650369 | 0.53732 | 0.793807 |
|  | Δ≥0.2 | difference | 535 | 1631 | 1457 |
|  |  | no difference | 296 | 1341 | 415 |
|  |  | precision | 0.643803 | 0.548789 | 0.778312 |
| pvalu≤0.005 | Δ≥0.1 | difference | 1244 | 3714 | 3025 |
|  |  | no difference | 703 | 3450 | 996 |
|  |  | precision | 0.638932 | 0.518425 | 0.7523 |
|  | Δ≥0.15 | difference | 1017 | 2547 | 2258 |
|  |  | no difference | 589 | 2192 | 753 |
|  |  | precision | 0.63325 | 0.537455 | 0.749917 |
|  | Δ≥0.2 | difference | 759 | 1639 | 1531 |
|  |  | no difference | 443 | 1341 | 550 |
|  |  | precision | 0.631448 | 0.55 | 0.735704 |
| pvalue≤0.01 | Δ≥0.1 | difference | 1446 | 3720 | 3138 |
|  |  | no difference | 863 | 3425 | 1194 |
|  |  | precision | 0.626245 | 0.520644 | 0.724377 |
|  | Δ≥0.15 | difference | 1165 | 2549 | 2312 |
|  |  | no difference | 711 | 2173 | 893 |
|  |  | precision | 0.621002 | 0.539814 | 0.721373 |
|  | Δ≥0.2 | difference | 858 | 1640 | 1558 |
|  |  | no difference | 533 | 1328 | 634 |
|  |  | precision | 0.616822 | 0.552561 | 0.710766 |
| pvalue≤0.05 | Δ≥0.1 | difference | 2021 | 3724 | 3364 |
|  |  | no difference | 1430 | 3476 | 1764 |
|  |  | precision | 0.585627 | 0.517222 | 0.656006 |
|  | Δ≥0.15 | difference | 1579 | 2550 | 2422 |
|  |  | no difference | 1113 | 2198 | 1266 |
|  |  | precision | 0.586553 | 0.537068 | 0.656725 |
|  | Δ≥0.2 | difference | 1101 | 1641 | 1601 |
|  |  | no difference | 785 | 1342 | 856 |
|  |  | precision | 0.583775 | 0.550117 | 0.651608 |

**
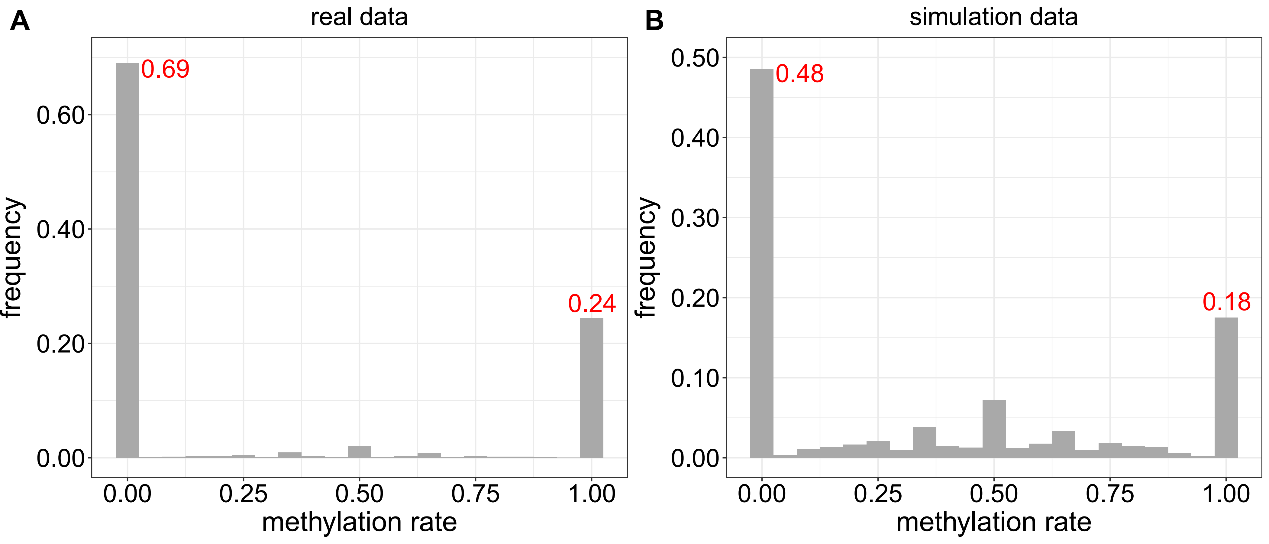
**

**Figure S1. Comparing the Distribution of Simulated and Real Data. The red values in the figure represent the observed methylation rates at 0 and 1, respectively.**


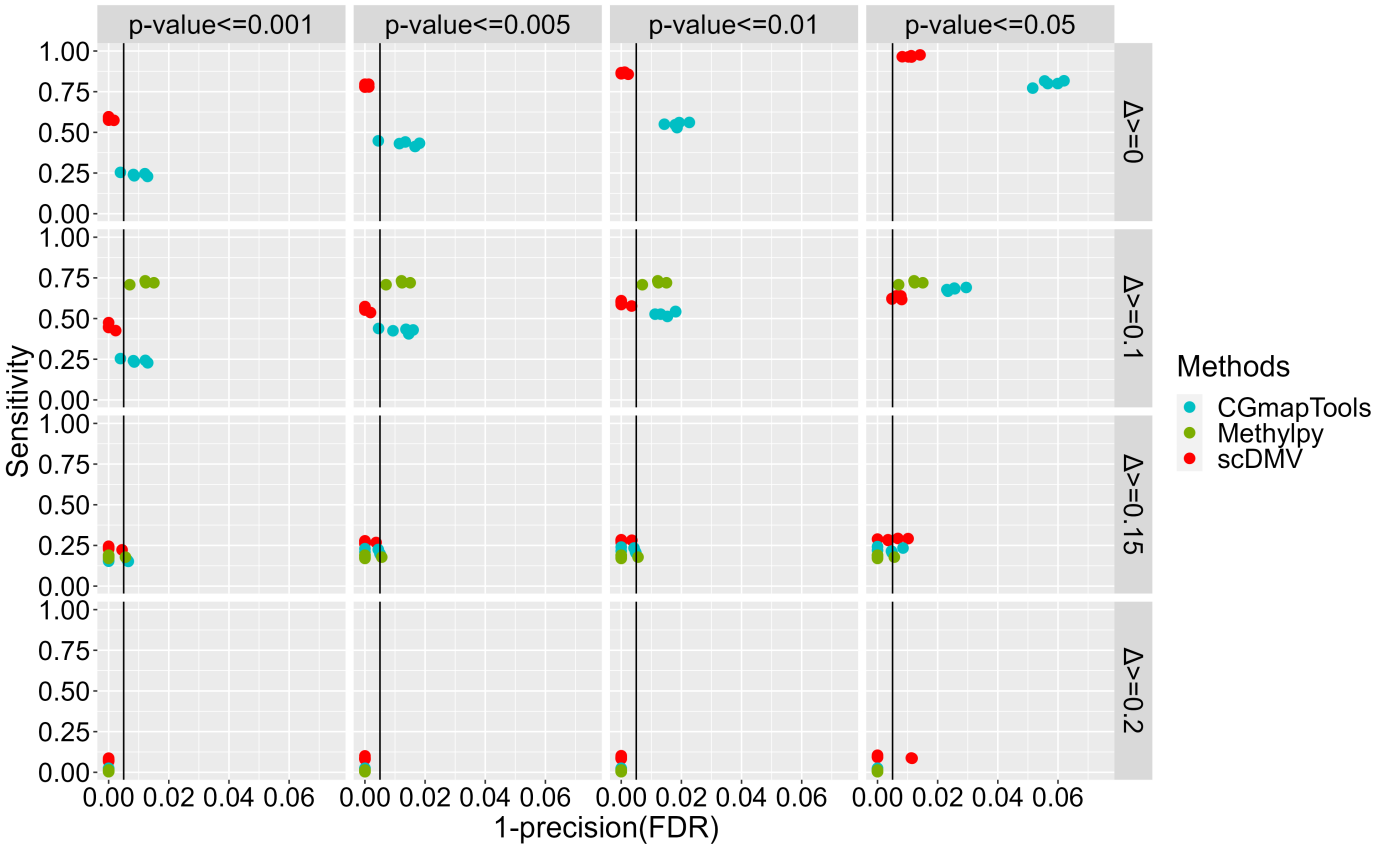


**Figure S2. Plotting Sensitivity on FDR: Results from Five Simulation Experiments**


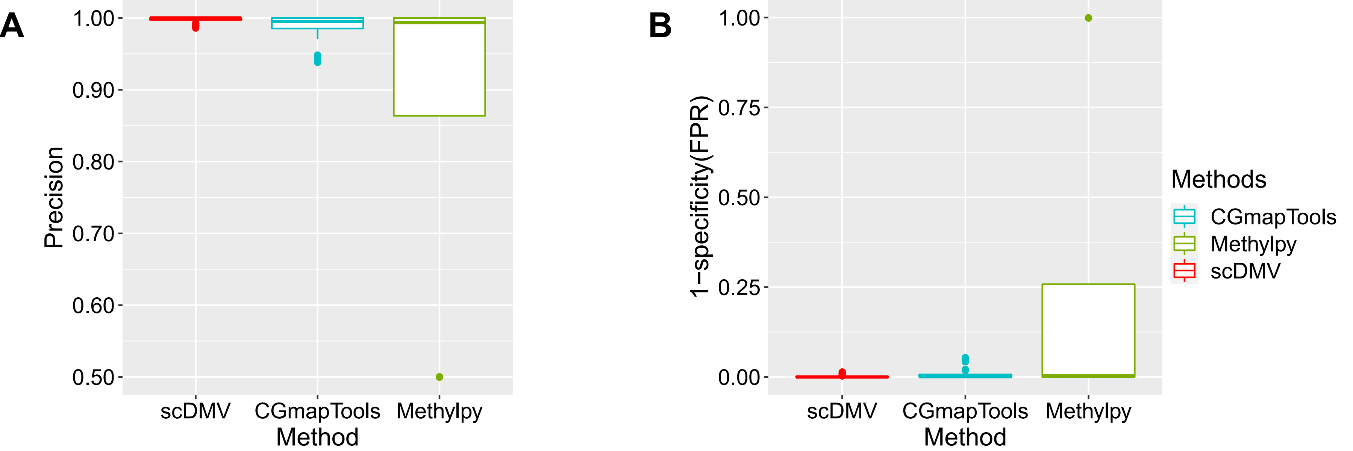


**Figure S3. Precision and False Positive Rate of the Three Methods**
