# Supplementary figures and images for: scDMV: a zero–one inflated beta mixture model for DNA methylation variability with scBS-seq data
Source: Bioinformatics. 2023 Dec 23;40(1):btad772. doi: 10.1093/bioinformatics/btad772 (PMC10786675; doi:10.1093/bioinformatics/btad772)

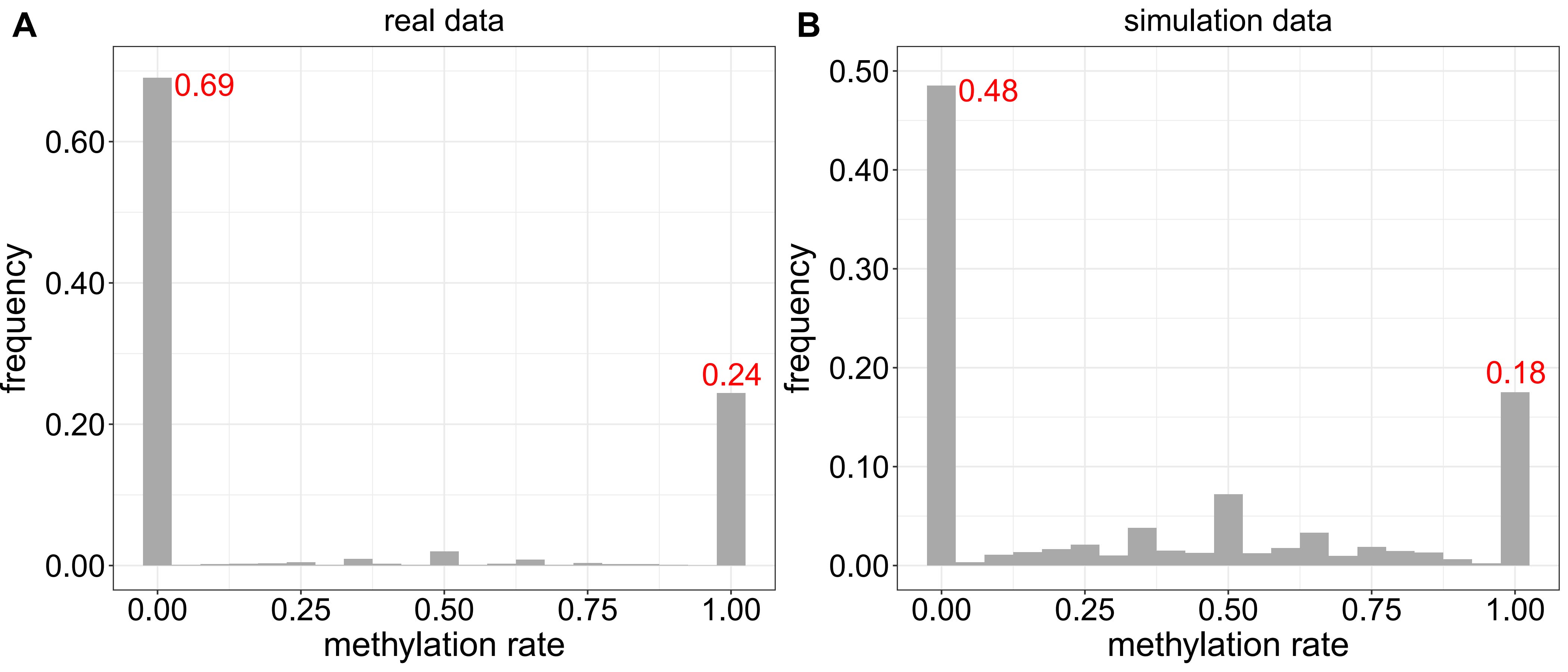

Supplement: btad772_Supplementary_Data [file btad772_supplementary_data.zip › figureS1-300dpi.jpg]

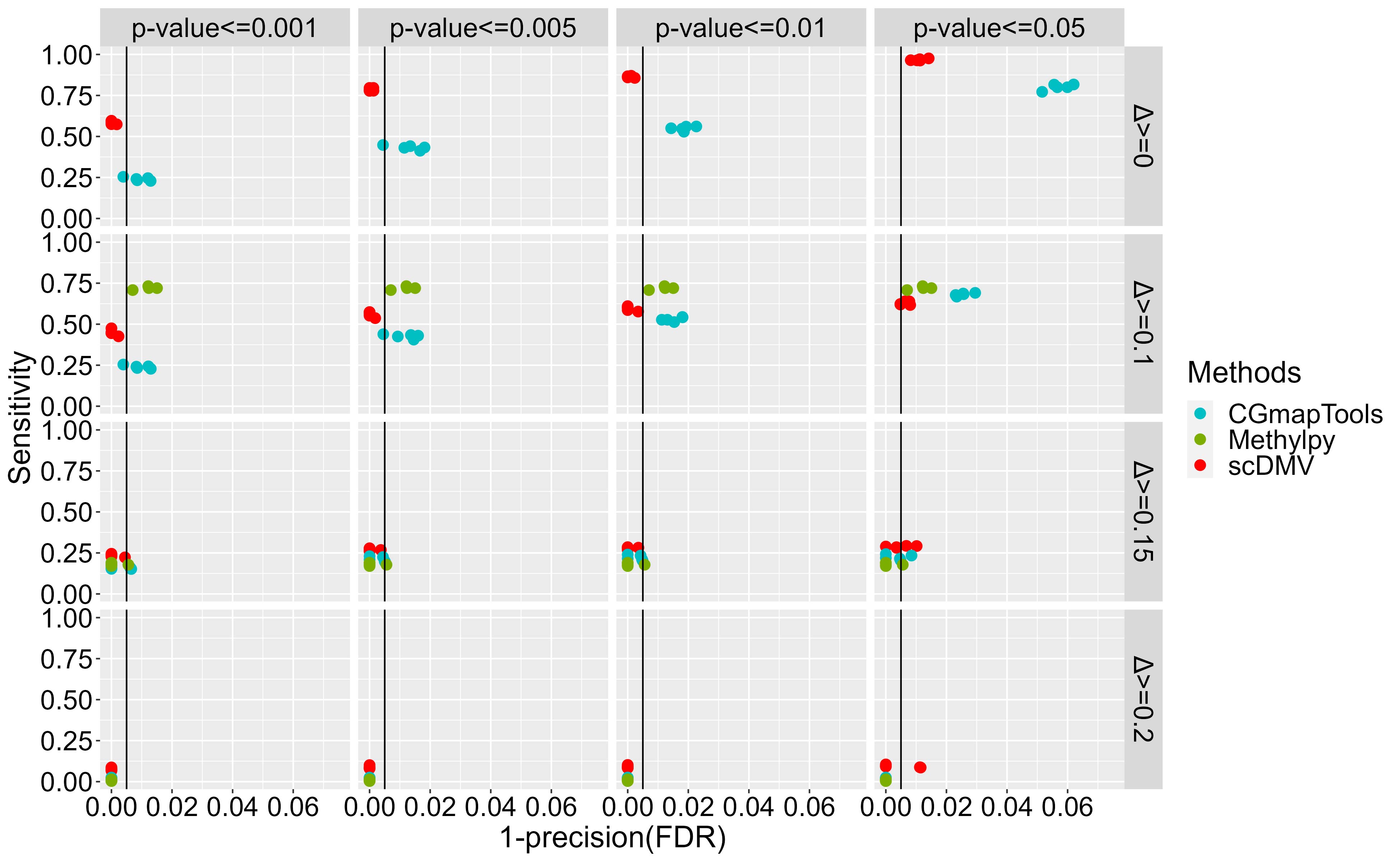

Supplement: btad772_Supplementary_Data [file btad772_supplementary_data.zip › figureS2-300dpi.jpg]

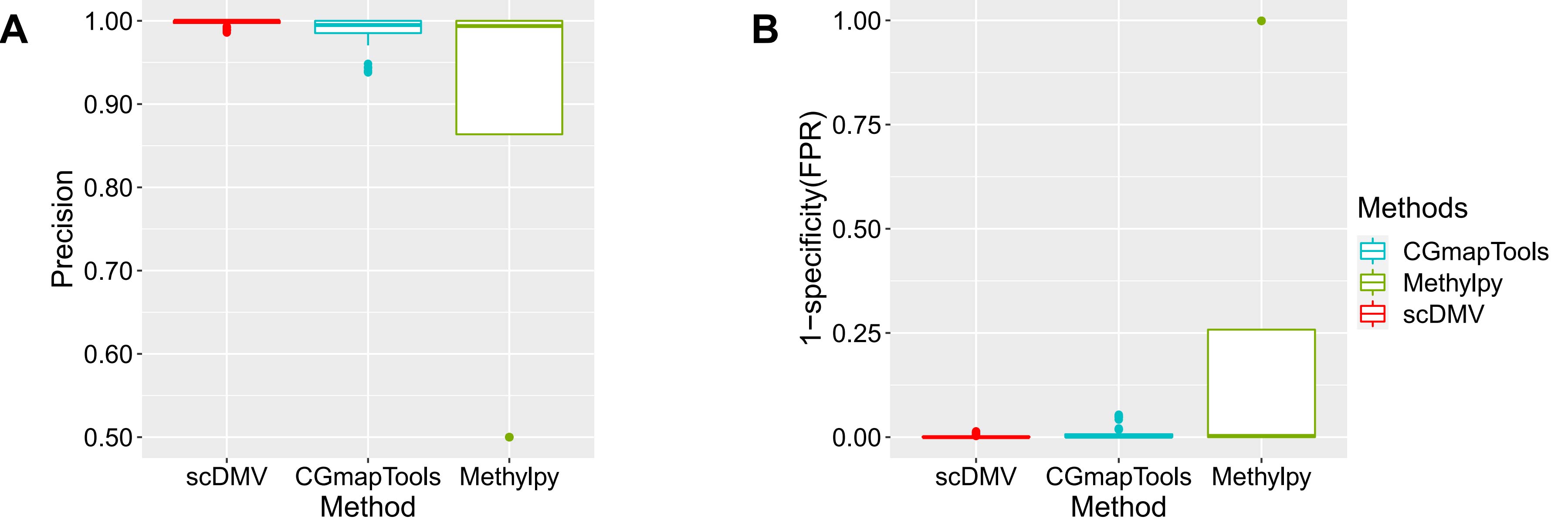

Supplement: btad772_Supplementary_Data [file btad772_supplementary_data.zip › figureS3-300dpi.jpg]
